# Band structures of passive films on titanium in simulated bioliquids determined by photoelectrochemical response: principle governing the biocompatibility

**DOI:** 10.1080/14686996.2022.2066960

**Published:** 2022-05-06

**Authors:** Seong-Cheol Kim, Takao Hanawa, Tomoyo Manaka, Hiroaki Tsuchiya, Shinji Fujimoto

**Affiliations:** a Division of Materials and Manufacturing Science, Graduate School of Engineering, Osaka University, Suita, Osaka, Japan; b Institute of Biomaterials and Bioengineering, Tokyo Medical and Dental University (TMDU), Tokyo, Japan; c Center for Advanced Medical Engineering Research and Development, Kobe University, Kobe, Japan; d Graduate school of Medical and Dental Sciences, Tokyo Medical and Dental University (TMDU), Tokyo, Japan

**Keywords:** band gap, band structure, biocompatibility, Hanks’ solution, passive film, photocurrent, saline, Titanium, XPS

## Abstract

The band structures and band gap energies, *E*_g_, of passive films formed on titanium (Ti) in simulated bioliquids, Hanks’ solution (Hanks) and saline, were evaluated. Ti was polarized at 0, −0.1, and −0.2 V_Ag/AgCl_, *E*_f_, for 1 h. After polarization, the surfaces were characterized using X-ray photoelectron spectroscopy, and the photoelectrochemical responses were evaluated. The current change during photoirradiation was recorded as a photocurrent transient at each measuring potential, *E*_m_, and by changing the wavelength of the incident light. Passive films consisted of a very thin TiO_2_ layer containing small amounts of Ti_2_O_3_ and TiO, hydroxyl groups, and water. During polarization in Hanks, calcium and phosphate ions were incorporated or formed calcium phosphate but not in saline. Calcium phosphate and hydroxyl groups influenced the band structure. *E*_g_ was graded in Hanks but constant in saline, independent of *E*_f_ and *E*_m_. The passive film on Ti behaved as an *n*-type semiconductor containing two layers: an inner oxide layer with a large *E*_g_ and an outer hydroxide layer with a small *E*_g_. In Hanks, *E*_g_ was 3.3–3.4 eV in the inner oxide layer and 2.9 eV in the outer hydroxide layer. In saline, *E*_g_ was 3.3 eV in the inner layer and 2.7 eV in the outer layer. Calcium phosphate and hydroxyl groups influenced the band structure of the passive film. The *E*_g_ of the outermost surface was smaller than that of TiO_2_ ceramics, which is probably one of the principles of the excellent biocompatibility of Ti among metals.

## Graphical abstract

**Figure:**
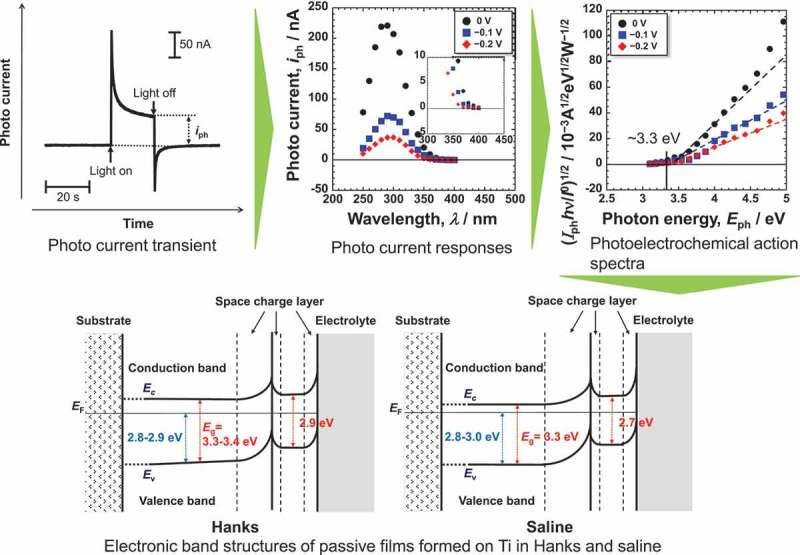

## 1. Introduction

Titanium (Ti) and its alloys are widely used as implant devices in medicine and dentistry because of their excellent corrosion resistance and high specific strength [1]. Their good tissue compatibility is well established through significant evidence from basic research and high clinical performance. For example, in orthopedics, bone screws and bone nails consisting of Ti alloys usually form calluses and assimilate into bone tissue after long-term implantation, inducing refracture of the bone during retrieval [2]. This is because of the good compatibility of Ti alloys with hard tissues. Many studies on the hard-tissue compatibility of Ti have been performed, and detailed information can be found in the literature [1,3]. The initial reaction at the interface directly influences the biocompatibility of the material. Therefore, biocompatibility is governed by the surface properties of the material. However, the principle and mechanism of the good tissue compatibility of Ti among metals have not been completely elucidated, despite numerous studies conducted on biological reactions.

The Ti metal surface is covered by a passive oxide film, which contributes to its excellent corrosion resistance. The passive film on Ti is mainly an extremely thin amorphous TiO_2_ containing small amounts of Ti_2_O_3_ and TiO, along with water and hydroxyl groups [4–7]. In addition, as the composition is graded, more Ti^4+^ and OH^−^ ions appear near the surface of the film [6]. This passive film formation process has been discussed elsewhere [8], and the chemical state of the passive oxide film has also been precisely investigated [9]. It has been established that the composition, structure, and chemical state of the passive film are different from those of the crystalline TiO_2_ ceramics. For example, the adsorption kinetics of calcium and phosphate ions in passive films on Ti differ from those in TiO_2_ ceramics [10].

In this regard, the band gap energy, *E*_g_, between the valence and conduction bands of TiO_2_ crystalline ceramics is usually evaluated by the optical absorption edge. It is well known that the reactivity of TiO_2_ ceramics is governed by *E*_g_, and continuous efforts to decrease *E*_g_ have been made to activate their photocatalytic performance [11]. The *E*_g_ of a photocatalyst decreases as its optical response shifts to longer excitation wavelengths. From this viewpoint, the passive film on Ti already contains oxygen defects because of its nonstoichiometric composition. Therefore, the difference in the surface properties of the passive films on Ti and TiO_2_ ceramics is probably due to the difference in their *E*_g_s. The *E*_g_ of passive films on Ti after anodic oxidation and thermal oxidation has been investigated by the photoelectrochemical response in borate buffer solution, artificial seawater, and sulfuric acid [12–14] because the conventional techniques employed for oxide ceramics, such as ultraviolet absorption, cannot be used for thin passive films on Ti.

In this study, the *E*_g_ values of passive films formed on Ti in simulated bioliquids, Hanks’ solution and 0.9% NaCl aqueous solution, were evaluated using the photoelectrochemical response at potentials as close as possible to the open circuit potential (OCP). In addition, X-ray photoelectron spectroscopy (XPS) was performed to understand the effect of the chemical composition and chemical state of the passive films on the photoelectrochemical properties. This research will enhance our understanding of the properties of passive films on Ti in the human body.

## 2. Experimental procedure

### 2.1 Specimen

Commercially or industrially pure titanium (ISO grade 2; >99.5% Ti; Test Materials, Tokyo, Japan) rods (8 mm in diameter) were cut into disks (1.5 mm in thickness). In the case of OCP measurement and XPS, the disks were polished with SiC paper, followed by mirror finishing with a 0.04 μm SiO_2_ suspension. After polishing, the Ti disks were ultrasonically cleaned twice in acetone and once in isopropanol for 10 min each. The Ti disks were immersed in ultrapure water for 24 h to stabilize the passive films. The disks were then fixed in a polytetrafluoroethylene holder with an o-ring, exposing an area of 0.278 cm^2^ to the electrolyte. For photoelectrochemical measurements, Ti was connected to a lead wire by soldering. The disks and connected parts were embedded in epoxy resin, and the surface was polished with SiC paper, followed by mirror finishing with a 0.25 µm diamond suspension. After polishing, the specimens were ultrasonically cleaned sequentially in acetone, ethanol, and deionized water for 10 min each and immediately used for subsequent experiments.

### 2.2 Electrolytes

Hanks’ solution without glucose (Hanks) was prepared using reagent-grade chemicals in ultrapure water. The composition of the Hanks was similar to that of extracellular fluid which is as follows: Na^+^ 142, K^+^ 5.81, Mg^2+^ = 0.811, Ca^2+^ = 1.26, Cl^–^ = 145, PO_4_^3–^ = 0.778, SO_4_^2–^ = 0.811, and CO_3_^2–^ = 4.17 (mmol/L). The pH of Hanks was 7.4 after preparation and did not change during the experimental acquisition at 37ºC. A 0.9 mass% NaCl solution (saline) was also prepared for comparison. The pH of saline is 6.4 just after preparation at 37ºC.

### 2.3 Open circuit potential change with time

The changes in OCP with time in Hanks and saline were measured using a Pt counter electrode and an Ag/AgCl reference electrode. The OCP was measured after 72 h of immersing the specimens in the electrolyte.

### 2.4 Photoelectrochemical response

The specimens were immersed in Hanks and saline for 10 min. The specimens were then polarized at film formation potentials, *E*_f_, of −0.2, −0.1, and 0 V versus Ag/AgCl electrode in Hanks and saline for 1 h to form stable passive films. These potentials were determined according to the change in the OCP for 72 h. The potentials were as close as possible to the OCP. The pH of the saline after polarization was 5.3–5.8.

The photoelectrochemical response of the passive films was investigated using an experimental equipment similar to that used in a previous study [15–18]. The photoelectrochemical responses were measured using a potentiostat connected to a low-pass filter with a threshold frequency of 4 Hz and a differential amplifier. Monochromatic light from a 150 W xenon arc lamp and a grating monochromator was irradiated onto Ti specimens in an electrochemical cell through a quartz window. Current changes during photoirradiation for 20 s were recorded as photocurrent transients at each *E*_f_. The measurement was performed at each measuring potential, *E*_m_, by decreasing *E*_f_ at regular intervals of 0.1 V with the wavelength of the incident light varying from 250 to 450 nm.

### 2.5 X-ray photoelectron spectroscopy

After polarization at each *E*_f_ in both Hanks and saline for 1 h, the Ti specimens were rinsed with ultrapure water to remove any chemical species not incorporated onto the surface and dried by a stream of N_2_ gas. Immediately after drying, the specimens were inserted in the pre-evacuation chamber of the XPS machine (JPS-9010MC, JEOL, Tokyo, Japan). Mg Kα line (1253.6 eV) was employed as the X-ray source. The binding energies were calibrated with the electron energy region of C 1s peak (285.0 eV) originating from the so-called contamination. The background of peaks was subtracted with Shirley’s method [19]. The composition and thickness of the passive film were simultaneously calculated according to previous studies [20,21] that is outlined elsewhere [7,22,23]. The results were statistically evaluated using one-way ANOVA with a significance of *p* < .05.

## 3. Results

### 3.1 Change in open circuit potential

**Figure 1.**
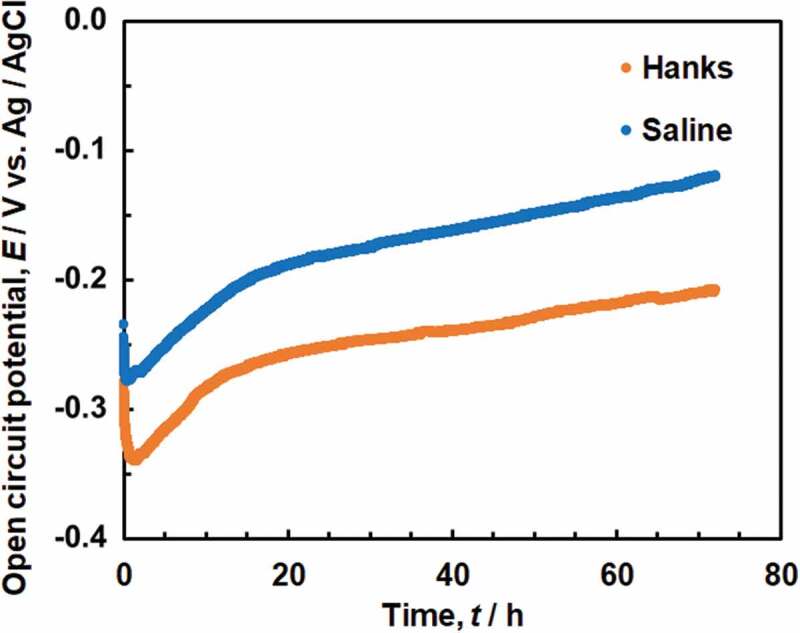
Change in open circuit potentials (OCP) of Ti in Hanks and saline for 72 h.

The pH of Hanks remained at 7.4 at 37ºC, and no precipitation was observed during the OCP measurement; the pH of saline gradually decreased to 5.7 after 6 h and 5.8 after 72 h at 37ºC. Changes in the OCP of Ti in Hanks and saline over time are shown in Figure 1. The OCP was between −0.2 V and −0.3 V in Hanks and saline just after starting the measurement and immediately decreased after immersion, followed by a gradual increase with time. After 72 h, the OCP was approximately −0.2 V in Hanks and −0.1 V in saline. The OCP in saline was always more noble than that in Hanks because the pH of saline was lower than that of Hanks.

### 3.2 Photoelectrochemical response

**Figure 2.**
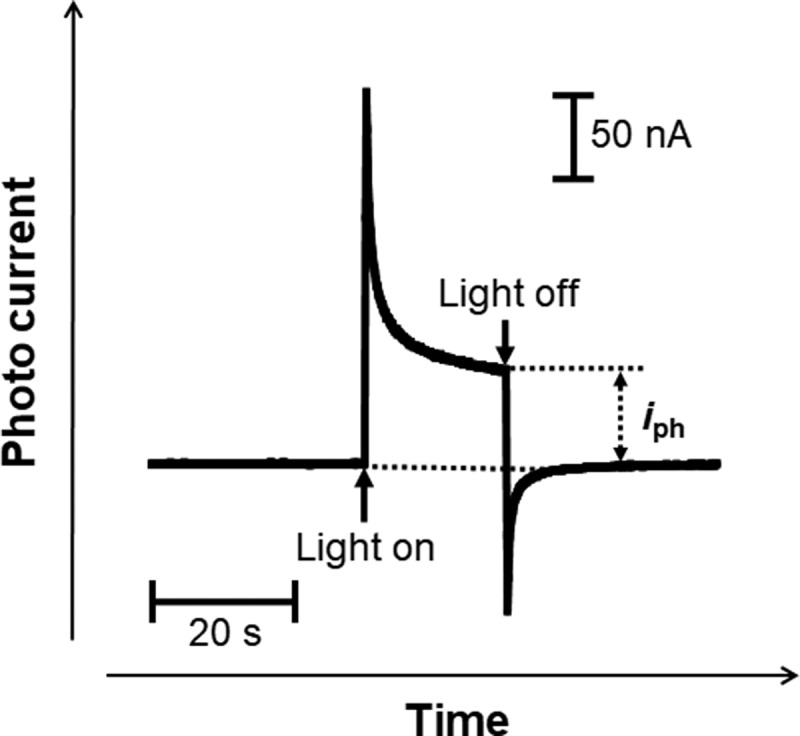
Example of photocurrent transient generated with the light on and off for passive films on Ti.

A typical photocurrent transient for passive films formed on Ti in Hanks and saline is shown in Figure 2. The shapes of the current transients in all the specimens were similar to that in this figure. The current varies during photoirradiation; therefore, the current recorded after 20 s was defined as the photocurrent, *i*_ph_, for analysis in the present work.

**Figure 3.**
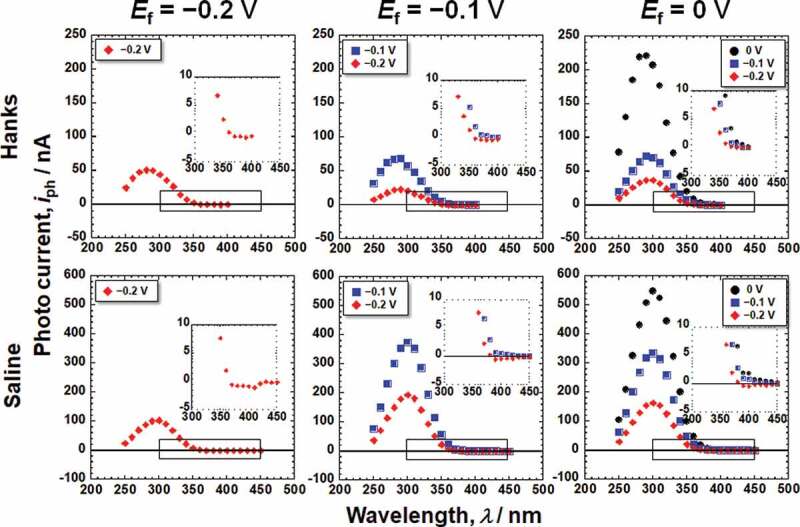
Photocurrent responses of the passive films formed on Ti in Hanks and saline.

**Figure 4.**
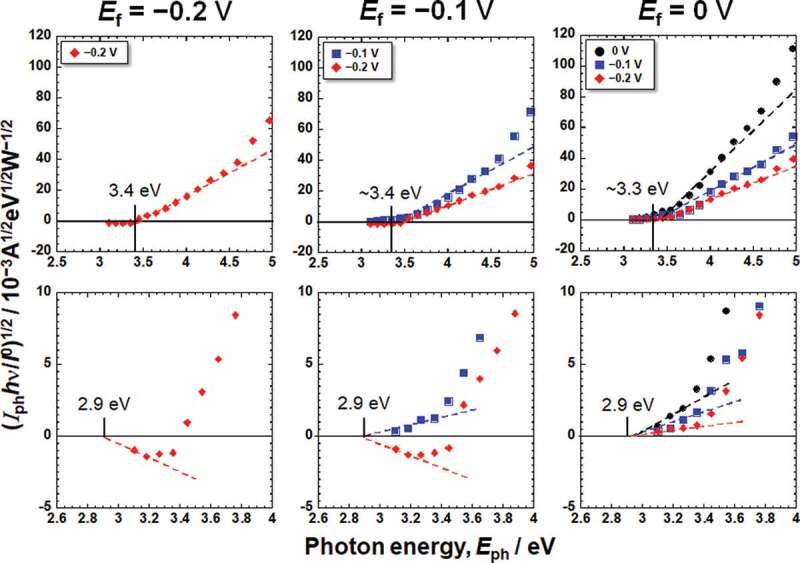
Photoelectrochemical action spectra calculated from the steady photocurrent in Hanks shown in Figure 3.

**Figure 5.**
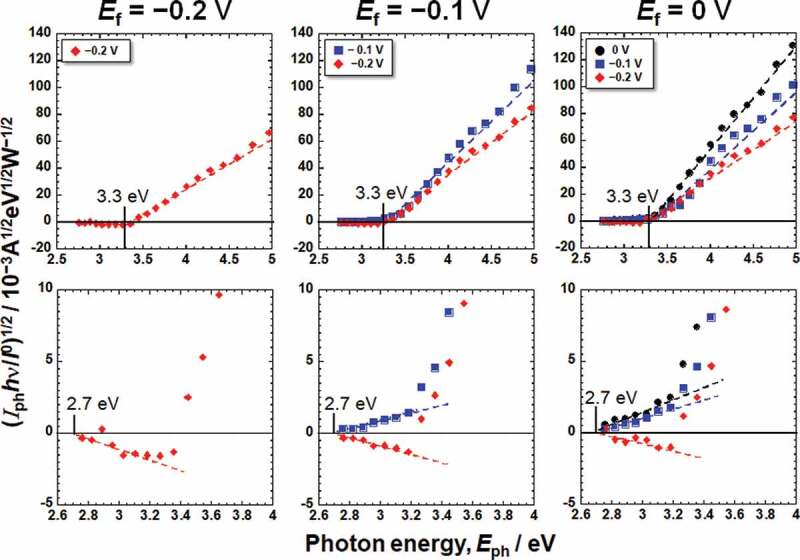
Photoelectrochemical action spectra calculated from the steady photocurrent in saline shown in Figure 3.

Figure 3 shows the photocurrent responses of the passive films formed on Ti in Hanks and saline. The photocurrent varied depending on *E*_m_ and the wavelength of the incident light. The photocurrent spectra were normalized for further analysis because the intensity of the light is not constant for each wavelength. Assuming that the photoexcitation is generated as an indirect transition, the photocurrent spectra were normalized as a photoelectrochemical action spectrum as follows [15–18].

(1) $$({i_{ph}}^{.}hv/I_{0}{)}^{1/2}=C(hv-E_{g}),$$

where *I*_0_ and *hν* are the intensity and photon energy of the incident light, respectively, *C* is the slope of the photoelectrochemical action spectrum and reflects the amplitude of the generated photocurrent, and *h* is Planck’s constant. By replotting the photocurrent spectra in Figure 3, photoelectrochemical action spectra against the photon energy of irradiated light, *E*_ph_, were obtained, as shown in Figures 4 and 5, corresponding to the Hanks and saline, respectively. The bottom figures show enlarged views of the lower photon energy region. In the case of Hanks, the photoelectrochemical action spectra did not exhibit a straight line with a constant *C*, instead *C* gradually changed. When the photocurrent is generated from a uniform composition layer, the direction of the photocurrent does not change at a fixed potential, even if the photon energy of the incident light is changed. In other words, *E*_g_ of the passive film was not uniform in the Hanks.

On subtracting the photoelectrochemical response of a component with lower band gap energy from the original photoelectrochemical action spectra, the photoelectrochemical action spectra for the other component with higher band gap energy are obtained in the higher photon energy region. The extrapolation of the remaining spectra on the horizontal axis represents a larger *E*_*g*_ component. The intersection between the extrapolation of the mean gradient lines and the horizontal axis represents *E*_g_. For Hanks, the larger component of *E*_g_ was in the range of 3.3–3.4 eV. *C* decreased near the lower photoelectrochemical action region, and the intersection shows that the *E*_g_ was 2.9 eV. This value was identical for all the examined specimens in Hanks. In addition, the value of *C* for the *E*_f_ of −0.2 V was negative. In the case of saline, the *E*_g_ was determined to be 3.3 eV. Near the lower photoelectrochemical action region, *E*_g_ was 2.7 eV. The band gap values in Hanks were larger than those in the saline.

### 3.3 X-ray photoelectron spectroscopy

**Figure 6.**
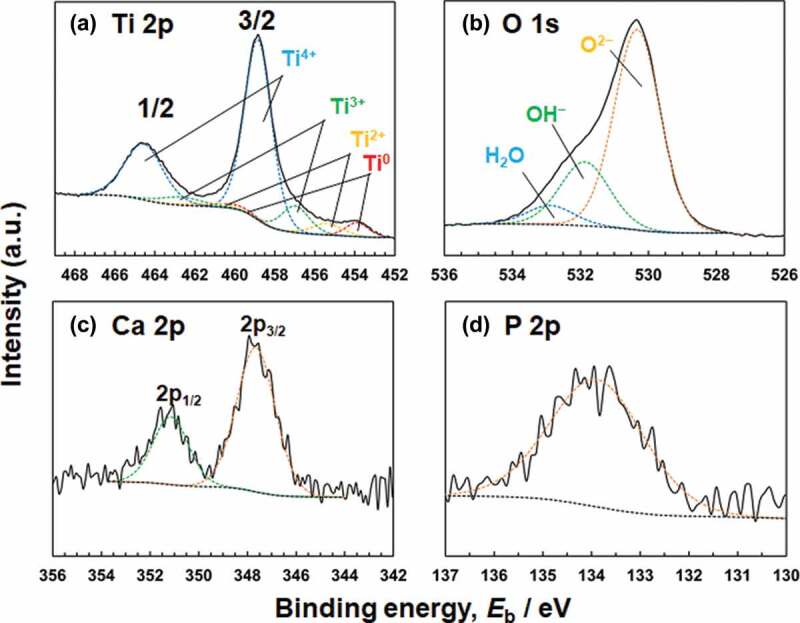
(a) Ti 2p, (b) O 1s, (c) Ca 2p, and (d) P 2p electron energy region spectra obtained from Ti after polarization at 0 V in Hanks for 1 h.

**Figure 7.**
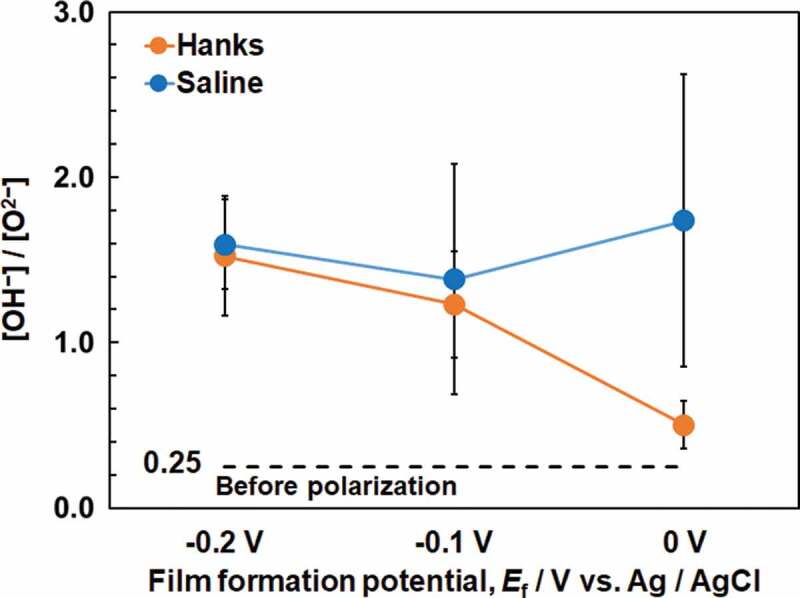
[OH^−^]/[O^2−^] ratios calculated form O 1s electron energy region spectra of Ti before and after polarization at each film formation potential, *E*_f_ (*n* = 3).

Titanium and oxygen were detected in all the Ti specimens. Carbon and nitrogen were also detected in the contaminated layer. In addition to the above elements, calcium and phosphorus were detected in specimens polarized in Hanks. No peak originating from CO_3_^2–^ was observed at approximately 289.6 eV [24]. The Ti 2p, O 1s, Ca 2p, and P 2p electron energy region spectra obtained from Ti polarized at an *E*_f_ of 0 V are shown in Figure 6. Figure 6 shows the deconvolution of the spectrum in the Ti 2p electron energy region. The Ti 2p spectrum contains four doublets corresponding to the metallic state of Ti^0^ and the oxide states of Ti^2+^, Ti^3+^, and Ti^4+^. The binding energy of each valence state of titanium was determined from the previously published data [5]. The binding energy of the peak originating from Ti^4+^ before polarization was 458.8 eV and after polarization, it was 458.8–459.0 eV in both Hanks and saline. In addition, the [Ti^4+^]/([Ti^2+^] + [Ti^3+^] + [Ti^4+^]) ratio, calculated from the integrated intensities of the component peaks, was almost constant at 0.78–0.82 before and after polarization in Hanks and saline. In Figure 6, the O 1s region spectrum contains three component peaks originating from oxide, O^2–^; hydroxyl groups or hydroxide, OH^–^; and adsorbed water or hydrate, H_2_O [25]. The OH^–^ peak included a peak from the phosphate oxygen, but the proportion of the latter was small. The [OH^–^]/[O^2–^] ratios are shown in Figure 7. The ratios increased after polarization and those were the same at −0.2 V both in Hanks and saline. The ratio in Hanks significantly decreased with the increase in *E*_f_, while that in saline was almost constant independent of *E*_f_. Therefore, the ratio in saline was significantly larger than that in Hanks at 0 V. The binding energy of the Ca 2p_3/2_ electron was 347.6–347.7 eV (Figure 6), indicating that calcium existed as Ca^2+^ [26, 27]. The binding energy of the P 2p region peak was 133.9–134.2 eV, indicating that phosphorus existed as phosphate (Figure 6) [22,28]. Therefore, it can be concluded that the calcium and phosphorus ions exist in the passive film as calcium and phosphate ions and sometimes form calcium phosphate on the passive film.

The relative concentrations of Ti, O, Ca, and P in the passive film without the Ti substrate were calculated by assuming that the total amount of these elements was 100 at.%, as presented in Table 1. It is evident that after polarization in Hanks, calcium and phosphate ions were incorporated into the passive film, but their amounts did not significantly change with increasing *E*_f_. This table also contains ratios of calcium concentration to phosphorus concentration, [Ca]/[P]. The [Ca]/[P] ratios slightly increased with *E*_f_, while no significant difference was observed. The thickness of the passive film increased slightly after polarization. The thickness in Hanks was comparable to or smaller than that in saline, despite the incorporation of calcium and phosphate ions or the formation of calcium phosphate.

**Table 1.** Relative concentrations of elements, [Ca]/[p] ratios, and thickness of the passive film formed on Ti (*n* = 3)

Electrolyte	Film formation potential, *E*_f_/V	Relative concentration (at.%)				[Ca]/[P]	Thickness, *d*/nm
		Ti	O	Ca	P		
Before polarization		28.8 ± 1.2	71.2 ± 1.0	–	–	–	5.9 ± 0.2
Hanks	−0.2	13.2 ± 1.6	85.4 ± 2.1	0.5 ± 0.3	1.0 ± 0.4	0.5 ± 0.2	6.5 ± 0.2
	−0.1	14.4 ± 2.2	84.1 ± 2.2	0.6 ± 0.2	1.0 ± 0.1	0.6 ± 0.3	6.4 ± 0.4
	0	22.8 ± 1.5	76.1 ± 1.9	0.5 ± 0.1	0.7 ± 0.2	0.7 ± 0.2	6.3 ± 0.1
Saline	−0.2	14.1 ± 2.4	85.9 ± 2.4	–	–	–	6.4 ± 0.4
	0.1	11.0 ± 2.5	89.0 ± 2.5	–	–	–	6.9 ± 0.4
	0	13.5 ± 6.5	86.5 ± 6.5	–	–	–	6.9 ± 0.6

**Figure 8.**
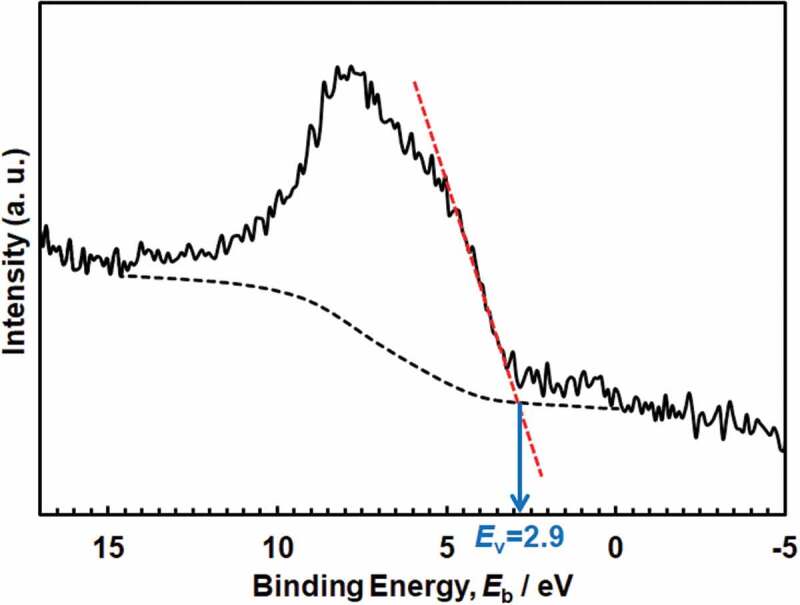
Valence band region spectra of Ti after polarization at 0 V in Hanks for 1 h and the determination of the maximum energy of valence band, *E*_v_.

The valence-band energy-region spectra are shown in Figure 8. The spectra were superimposed on those from the passive film and Ti substrate. In anatase, the valence band region contains two peaks at ~ 6 and ~8 eV, which correspond mainly to π (non-bonding) and σ (bonding) of O 2p orbitals, respectively [28]. The 3σ orbital of OH^−^ appears at a binding energy of ~10.8 eV [29]. The water molecule and 3σ orbital of the dissociated water lie at approximately 13 and 11 eV, respectively [30]. The maximum energy of the valence band, *E*_v_, against the Fermi energy, *E*_F_, was determined by linearly extrapolating the fermi-level-side slope of the valence band peak on the baseline [31]. The *E*_v_ was found to be 2.8–2.9 eV in Hanks and 2.8–3.0 eV in saline, while that in the polished Ti without polarization was 2.8 eV.

## 4. Discussion

XPS results revealed that the passive film on Ti consisted mainly of very thin TiO_2_ containing small amounts of Ti_2_O_3_ and TiO, along with hydroxyl groups and water (Figure 6). These results are in accordance with those of previous studies [5–7]. In addition, the composition is probably graded; more Ti^4+^ and OH^−^ exist near the surface of the film [6]. The passive film on Ti is non-stoichiometric TiO_2_ containing Ti_2_O_3_ and TiO that are electro conductive materials. However, the effect of Ti_2_O_3_ and TiO on the resultant *E*_g_ is unclear in this study. On the other hand, a large number of hydroxyl groups were detected using XPS. Therefore, similar to the passive film formed on Fe-Cr alloys, the passive film on Ti is assumed to consist of two layers [32]: inner oxide and outer hydroxide. After polarization, the [OH^−^]/[O^2−^] ratios increased in the passive films of both the Hanks and saline (Figure 7). The ratio in Hanks significantly decreased with the increase of *E*_f_, probably caused by the formation of calcium phosphate. As a result, relative concentration of O at 0 V in Hanks were significantly smaller than those under other conditions (Table 1) because of the decrease of OH^−^. The [OH^−^]/[O^2−^] ratios of the passive films on Co−28Cr−6Mo alloy (ASTM F799–95), Co–Ni–Cr–Mo alloy (ASTM F562), and 316 L-type stainless steel even before polarization were 2.9, 2.7, and 1.93 in a detection angle of 37°, respectively [33–35]. Therefore, the [OH^−^]/[O^2−^] ratio in the surface oxide film on Ti was much smaller than that on the Co–Cr alloys and 316 L-type stainless steel. The thickness of the passive film on Ti is much larger than that on Co–Cr alloys and 316 L-type stainless steel; thus, the relative thickness of the hydroxide layer and the relative amount of hydroxyl groups decrease.

The thickness of the passive film was increased by 0.4–1.0 nm by polarization (Table 1). As the concentrations of Na^+^ and Cl^−^ were almost the same in Hanks and saline, and other elements contained in Hanks were not contained in saline, the difference between the passive films formed in Hanks and those formed in saline may be due to the incorporation of calcium and phosphate ions or the formation of calcium phosphate in Hanks.

The *E*_g_ of the passive film formed in Hanks, 3.3–3.4 eV, was comparable or slightly larger than that in saline, 3.3 eV, independent of *E*_f_ and *E*_m_ (Figures 4 and 5). Interestingly, the *E*_g_s of the outer layer were identical for all the *E*_f_ and *E*_m_ in each solution: 2.9 eV in Hanks and 2.7 eV in saline. Even in the lower photon energy region, *E*_g_ in Hanks was larger than that in saline. The *E*_g_ of hydroxyapatite was determined experimentally as >6 eV and theoretically as 4.95 eV [36], whereas other studies reported it as 5.4 eV [37] and 4.51 eV [38]. The *E*_g_ of α-tricalcium phosphate (TCP) is 4.89 eV, and that of β-TCP is 5.25 eV [38]. These values are much larger than those of TiO_2_. The formation of calcium phosphate in Hanks probably increases *E*_g_ of the inner and outer layers.

The *E*_g_ values of the passive film and the slope *C* of the photoelectrochemical action spectrum were obtained, as shown in Figures 4 and 5. As mentioned above, *E*_g_ of the outer layer is much smaller than that inside the passive film. The passive film on Ti behaved as an *n*-type semiconductor. However, the photocurrents at the lower photon energy region at the *E*_f_ of −0.2 V and −0.1 V showed negative values at low *E*_m_ (Figures 4 and 5). We assume that the outer layer, where the band gap is small, is an *n*-type semiconductor and that the outer layer generates space charge layers at both the film–electrolyte interface and the outer layer–inner layer interface. The photocurrent changed from positive to negative with decreasing measurement potential, *E*_m_, because the gradients of the two space-charge layers were opposite. This phenomenon has been observed in a passive film on an Fe–Cr alloy with a *p*-type inner layer and an *n*-type outer layer [18]. The same phenomenon was observed in this study, even though both the inner and outer layers were *n*-type. Further discussion is necessary for the comprehension of this observation.

The *E*_g_ of the passive film on Ti anodized in H_2_SO_4_ is 3.25 ± 0.05 eV [12], whereas that anodized in artificial seawater is 3.4–3.7 eV and that anodized in borate buffer solution is 3.1 eV. After thermal oxidation at 400°C for 1 h, the *E*_g_ of the passive film on Ti is 3.4–3.7 eV [14]. Meanwhile, the *E*_g_ values of the passive films formed in Hanks and saline in this study, 3.2–3.4 eV, were almost the same as those in previous studies. The *E*_g_ of TiO_2_ ceramics, rutile and anatase, are 3.0 eV and 3.2 eV, respectively. Therefore, *E*_g_ of the passive film formed during anodic oxidation is comparable to or larger than that of TiO_2_ ceramics, while in the outermost surface of the passive film, it is smaller than that of TiO_2_ ceramics.

**Figure 9.**
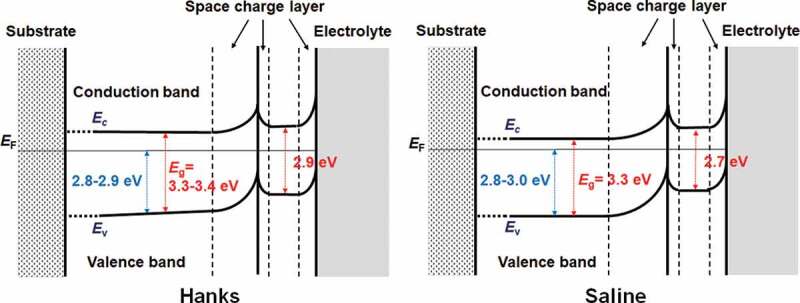
Electronic band structures of passive films formed on Ti in Hanks and saline.

From the above results and discussion, the electronic band structures of the passive film formed on Ti in Hanks and saline are shown in Figure 9. The *E*_g_ of the inner layer in Hanks was not constant but changed with depth because slope C was not constant but gradually decreased with decreasing photon energy (Figure 4). The band structure of the outer hydroxide layer was based on our previous study [16–18]. In the case of Hanks, *E*_g_ in the inner oxide layer was 3.3–3.4 eV. In the outermost surface layer, *E*_g_ was much lower (2.9 eV). In saline, *E*_g_ was 3.3 eV in the inner oxide layer and 2.7 eV in the outermost layer. In the inner oxide layer of Hanks, *E*_g_ was the same as or larger than that of saline, while *E*_v_, the energy difference between *E*_F_, the Fermi energy of the Ti substrate, and that of the valence band of the inner oxide layer, was 2.8–2.9 eV in Hanks and 2.8–3.0 eV in saline. Therefore, the minimum energy of the conduction band, *E*_c_, against *E*_F_ in Hanks was larger than that in saline.

Zr forms a highly stable and protective passive film, and the reactivity of Zr is much smaller than that of Ti [39]. In the case of the passive film on Zr, *E*_g_ is 3.01–3.47 eV in the outer hydroxide layer and 4.44–4.91 eV in the inner oxide layer [40]. The passive film on Zr consists mainly of ZrO_2_ with hydroxyl groups. The *E*_g_ is 4.27–4.93 eV by theory and 5.78–6.1 eV by experiment that varies according to the crystal systems [41]. These values are much larger than those for TiO_2_ and the passive film on Ti. Therefore, the reactivity of a material can be determined based on *E*_g_. The *E*_g_ values in the outermost layer are much smaller than those of TiO_2_ crystalline ceramics, rutile, and anatase, whereas the values in the inner layer are slightly larger than or almost the same as those of TiO_2_ ceramics. A very thin and non-stoichiometric composition probably reduces the *E*_g_. Therefore, differences in the composition, structure, and chemical state influence *E*_g_, and thinness and non-stoichiometric composition decrease *E*_g_. The decrease in th*e E*_g_ of a material activates its reactivity, as is known for TiO_2_ photocatalysts. The excellent biocompatibility of Ti among the metals may be induced by the low *E*_g_ of the outermost surface layer in the passive film, which has a high corrosion resistance. Calcium phosphate is regularly formed on Ti, but not on TiO_2_. Calcium phosphate formation kinetics on Ti are distinct from those on TiO_2_ crystalline ceramics [10]. The *E*_g_ values of the outermost surface of the passive film formed on Ti are smaller than those of the TiO_2_ ceramics, rutile (*E*_g_ = 3.0 eV), and anatase (*E*_g_ = 3.2 eV). Therefore, the Ti surface is more reactive with the surrounding environment than the TiO_2_ ceramic surface. This reactivity is probably one factor contributing to the excellent biocompatibility of Ti, in addition to its excellent corrosion resistance.

## 5. Conclusions

Passive films mainly consisted of a very thin TiO_2_ layer containing small amounts of Ti_2_O_3_ and TiO, hydroxyl groups, and water. During polarization in Hanks, calcium and phosphate ions were incorporated or formed calcium phosphate but not in saline. Calcium phosphate and hydroxyl groups influenced the band structure. *E*_g_ was graded in Hanks but constant in saline, independent of *E*_f_ and *E*_m_. The passive film on Ti behaved as an *n*-type semiconductor containing two layers: an inner oxide layer with a large *E*_g_ and an outer hydroxide layer with a small *E*_g_. In Hanks, the value of *E*_g_ in the inner layer was 3.3–3.4 eV, whereas it was much lower in the outermost surface layer (2.9 eV). In saline, *E*_g_ was 3.3 eV in the inner layer and 2.7 eV in the outermost layer. The *E*_g_ values of the outermost surfaces of the passive films formed on Ti were smaller than those of TiO_2_ ceramics. Therefore, the Ti surface is more reactive with the surrounding environment than the TiO_2_ ceramic surface. This is probably one of the reasons for the excellent biocompatibility of Ti among metals, in addition to its excellent corrosion resistance.

## Supplementary Material

Supplemental Material

Supplemental Material

Supplemental Material
