# Supplementary material for: Band structures of passive films on titanium in simulated bioliquids determined by photoelectrochemical response: principle governing the biocompatibility
Source: Sci Technol Adv Mater. 2022 May 6;23(1):322–31. doi: 10.1080/14686996.2022.2066960 (PMC9090409; doi:10.1080/14686996.2022.2066960)

Hanks_E_f 0 V_E_m 0 V

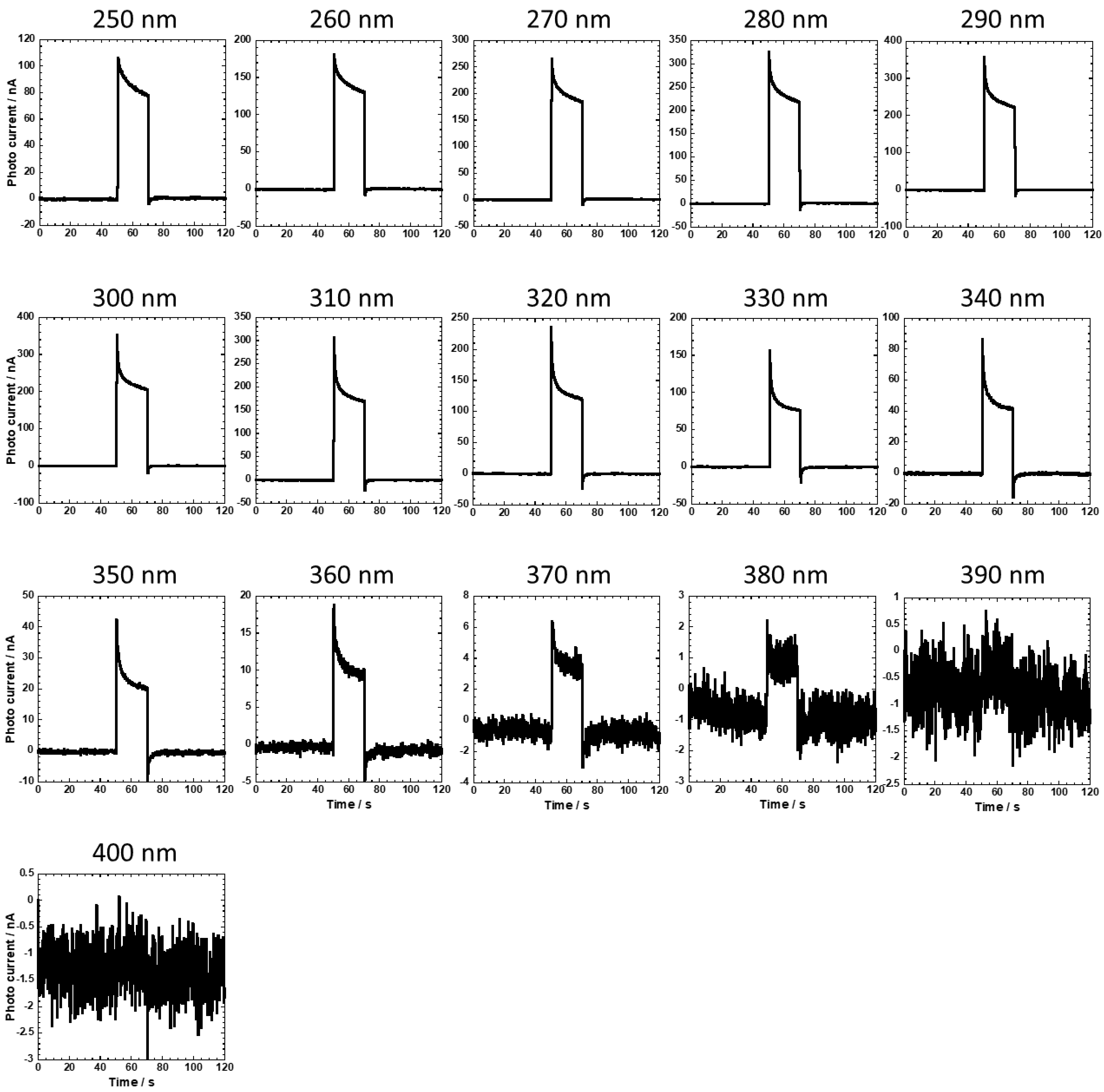

Hanks_E_f 0 V_E_m -0.1 V

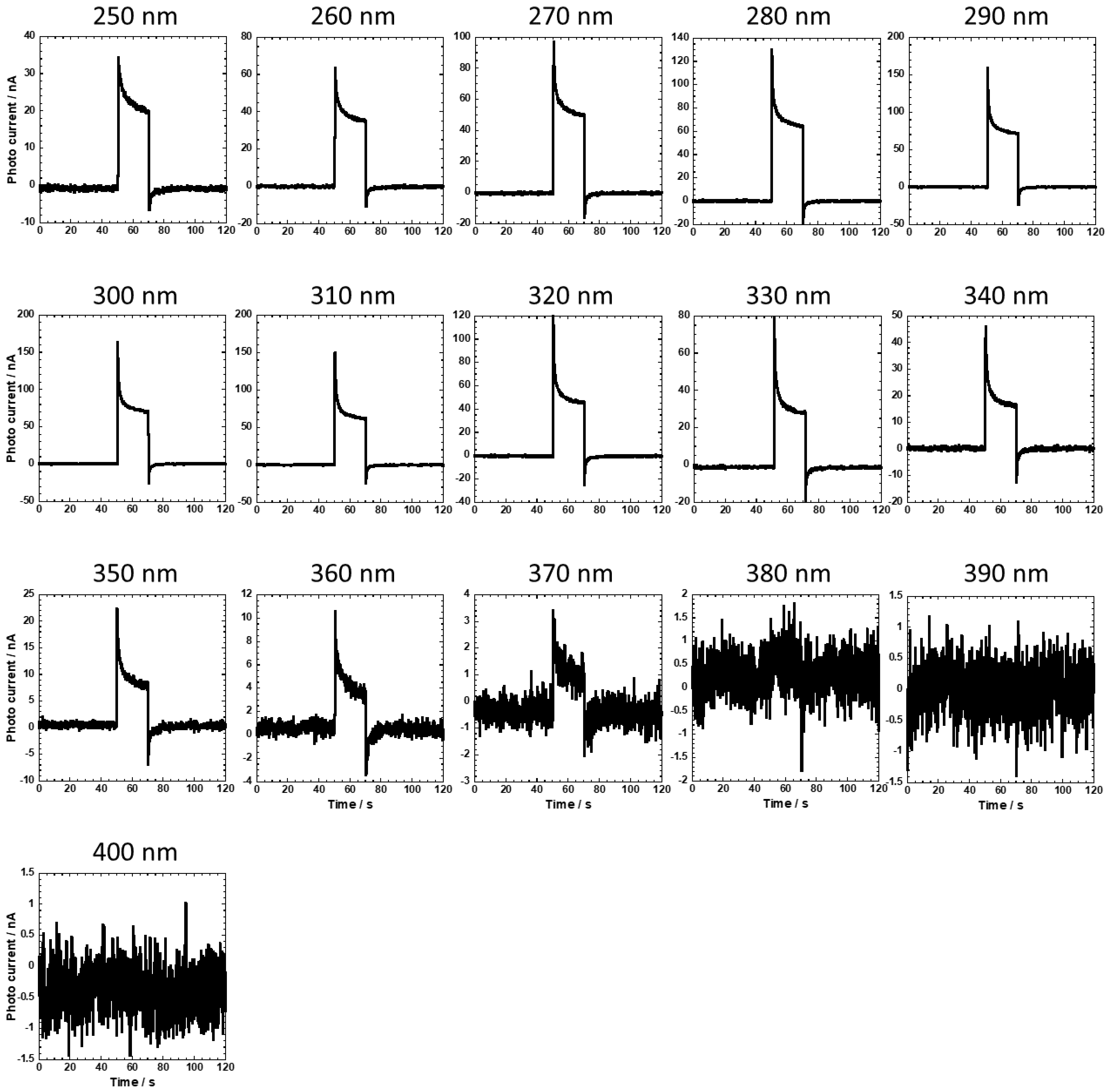

Hanks_E_f 0 V_E_m -0.2 V

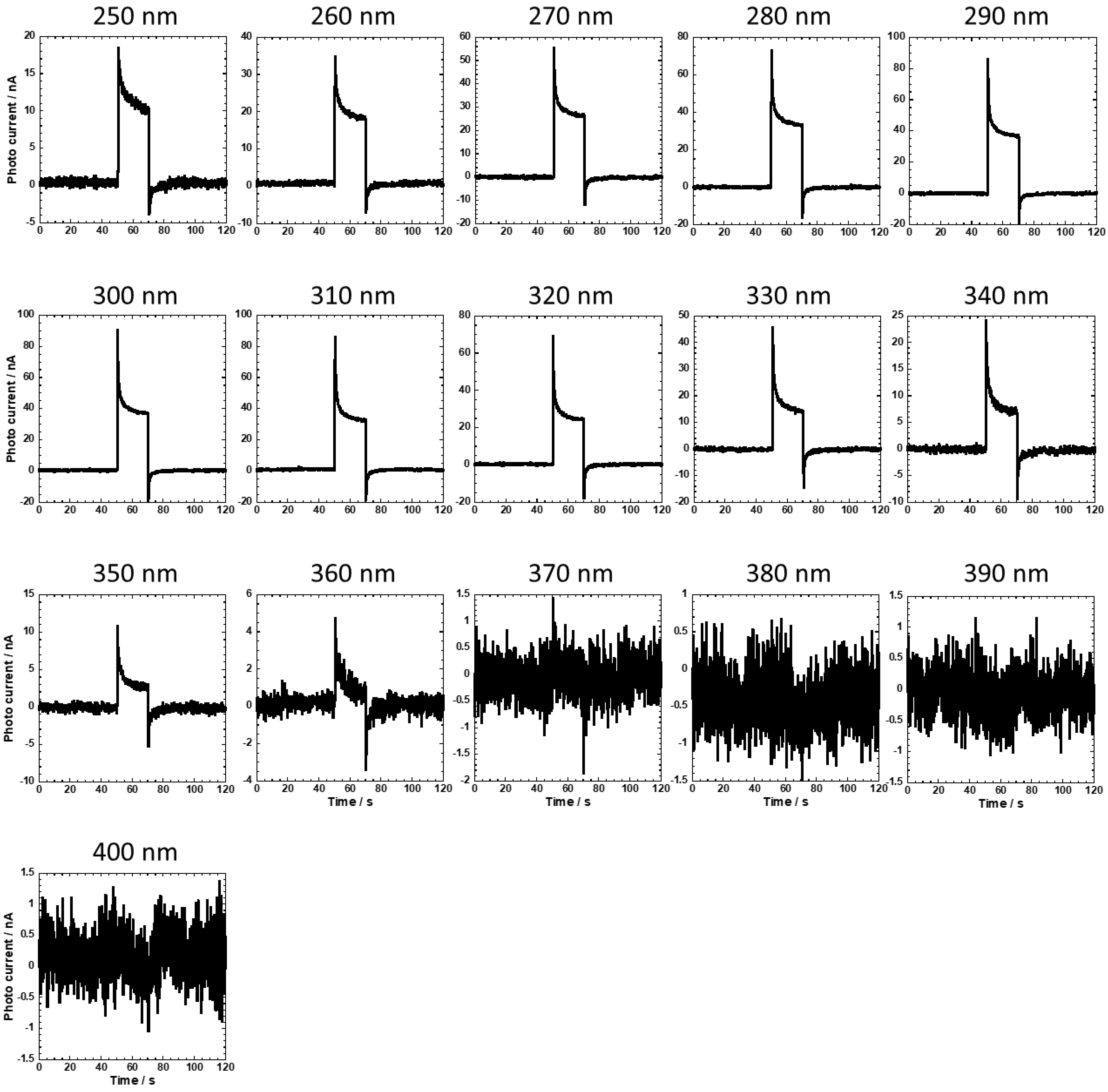

Hanks_E_f -0.1 V_E_m -0.1 V

250 nm

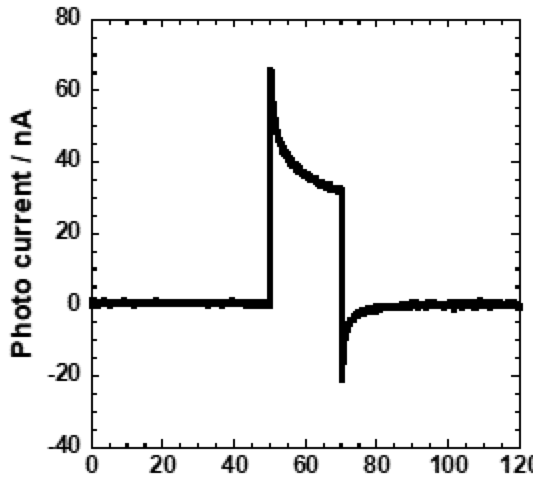

260 nm

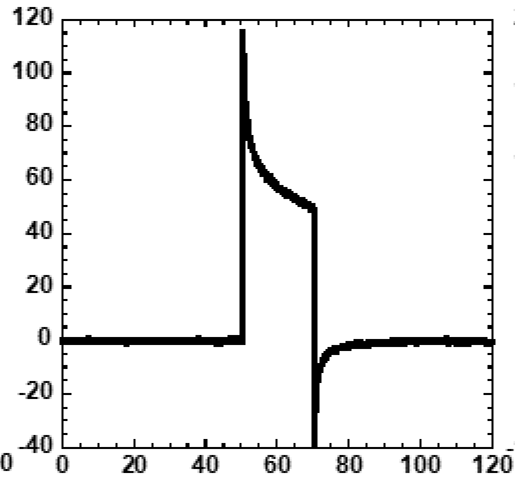

270 nm

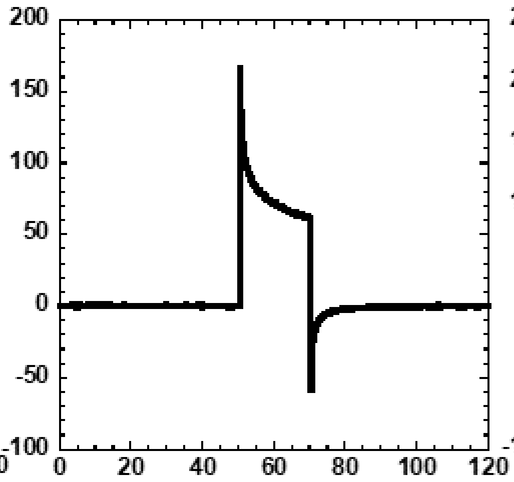

280 nm

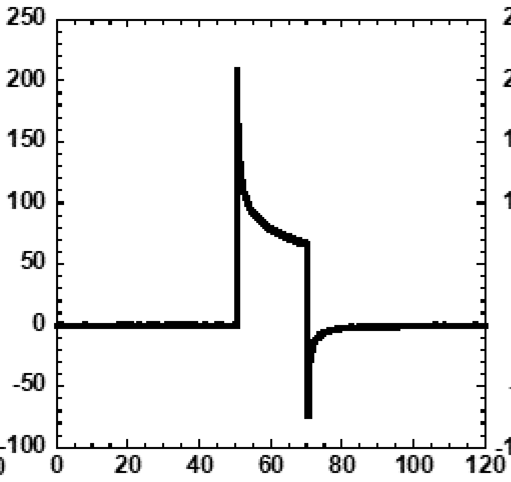

290 nm

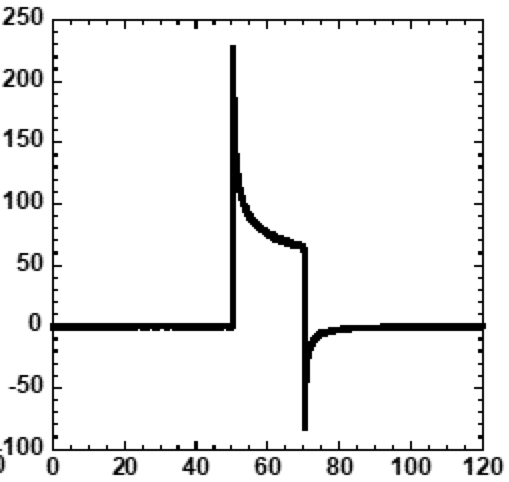

300 nm

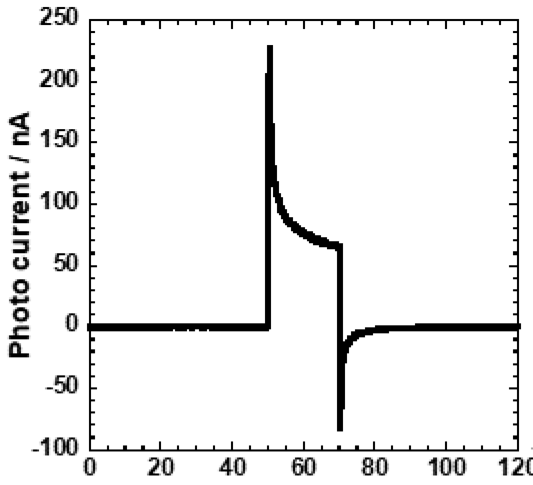

310 nm

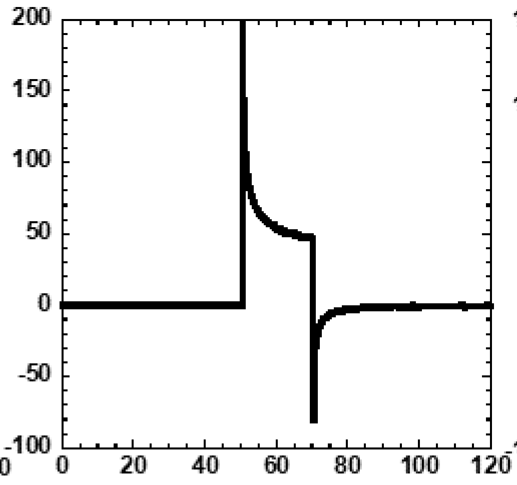

320 nm

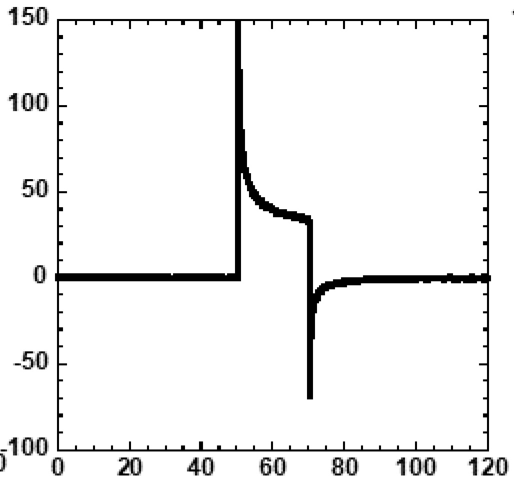

330 nm

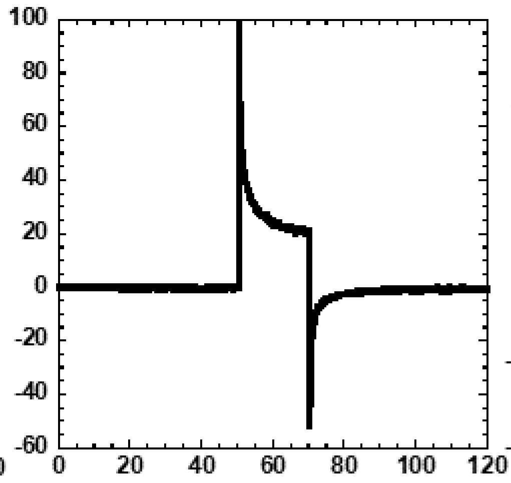

340 nm

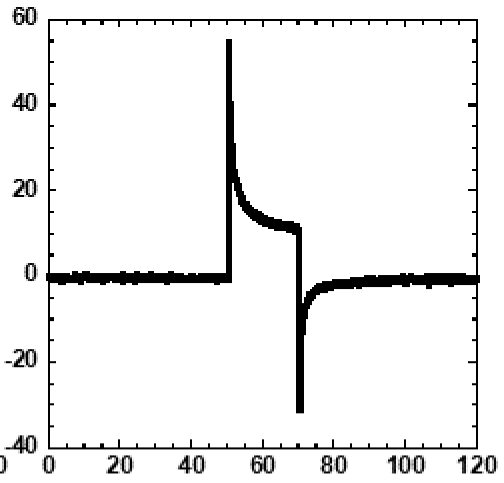

350 nm

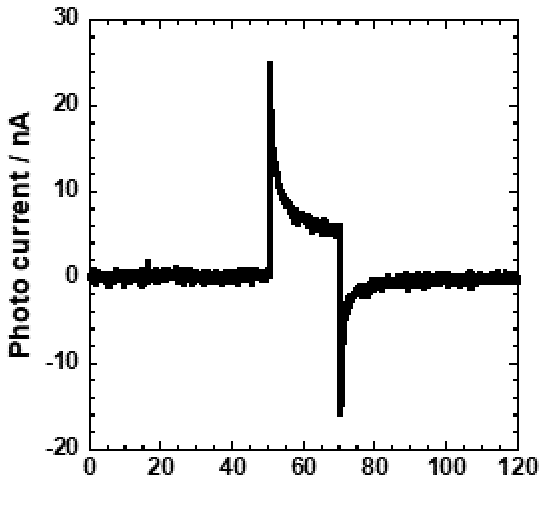

360 nm

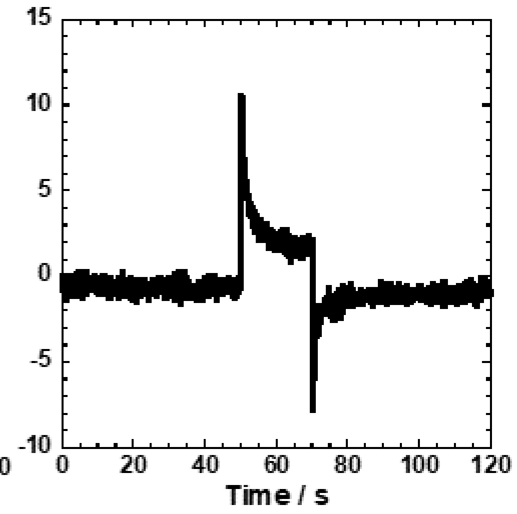

370 nm

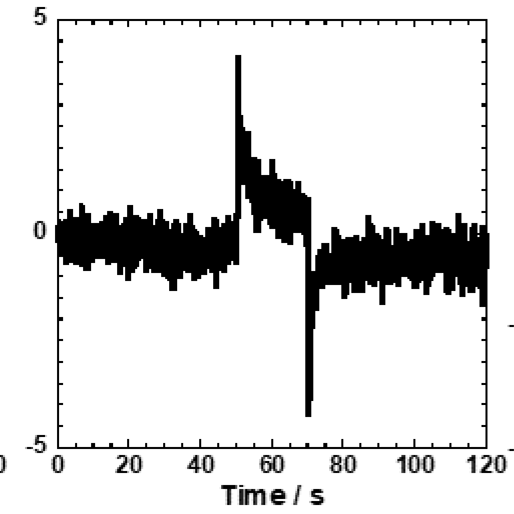

380 nm

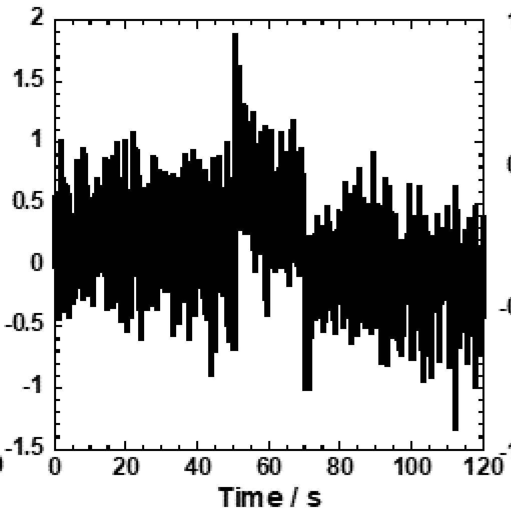

390 nm

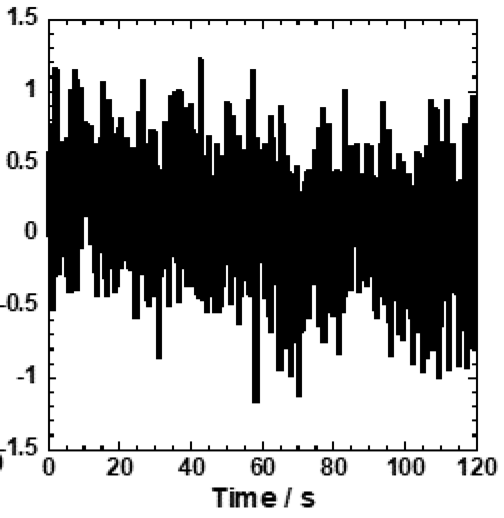

400 nm

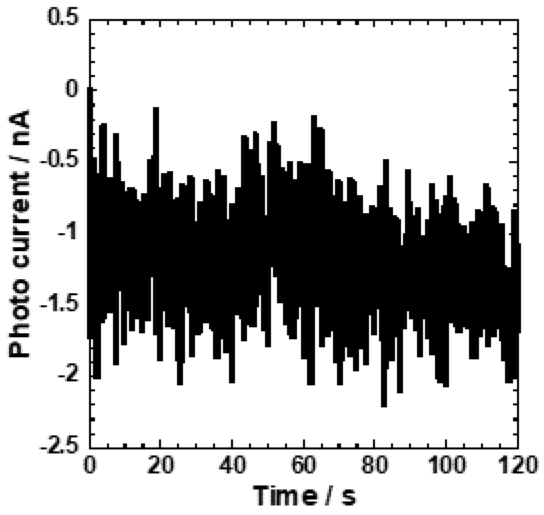

Hanks_E_f -0.1 V_E_m -0.2 V

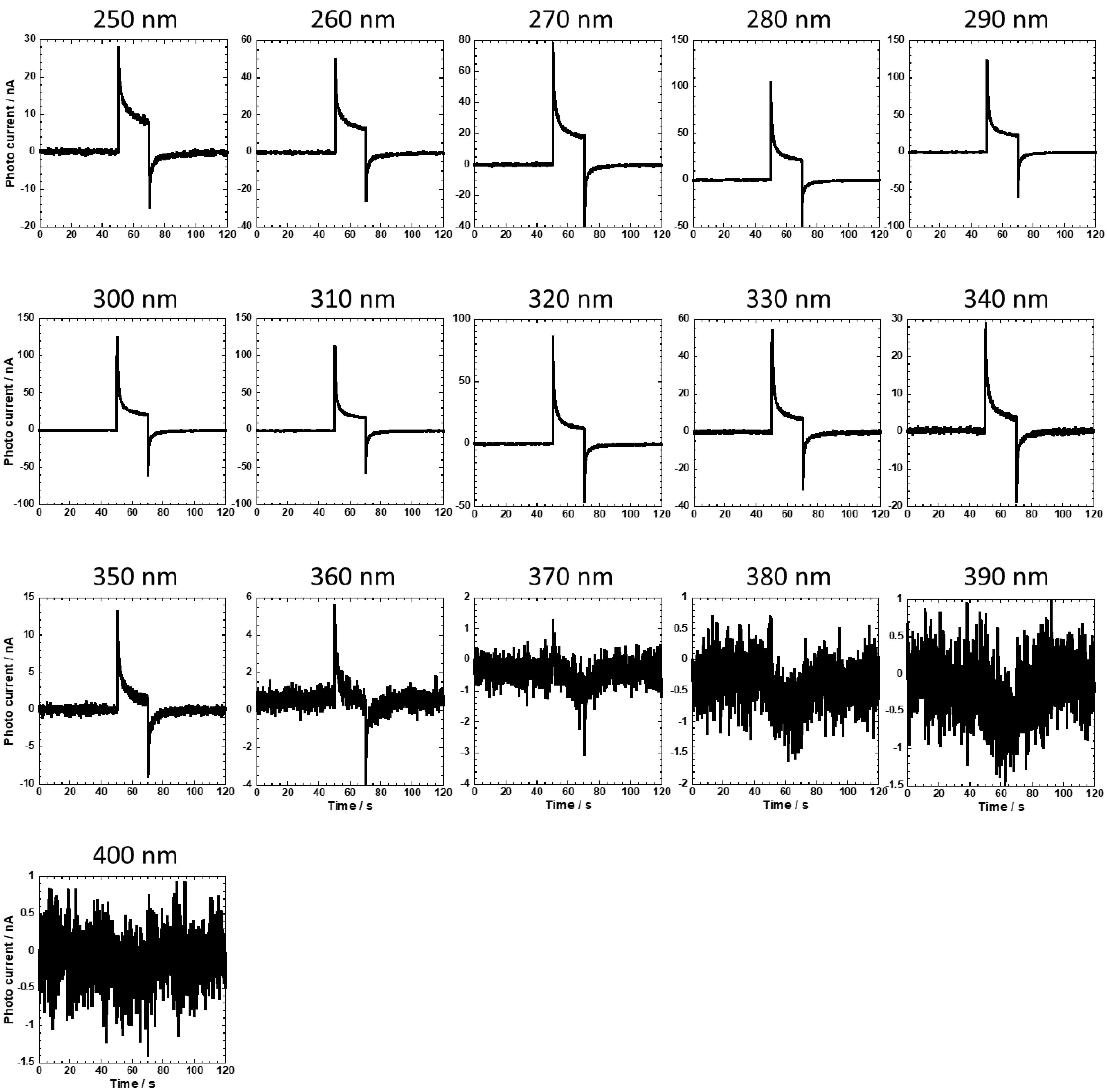

Hanks_E_f -0.2 V_E_m -0.2 V

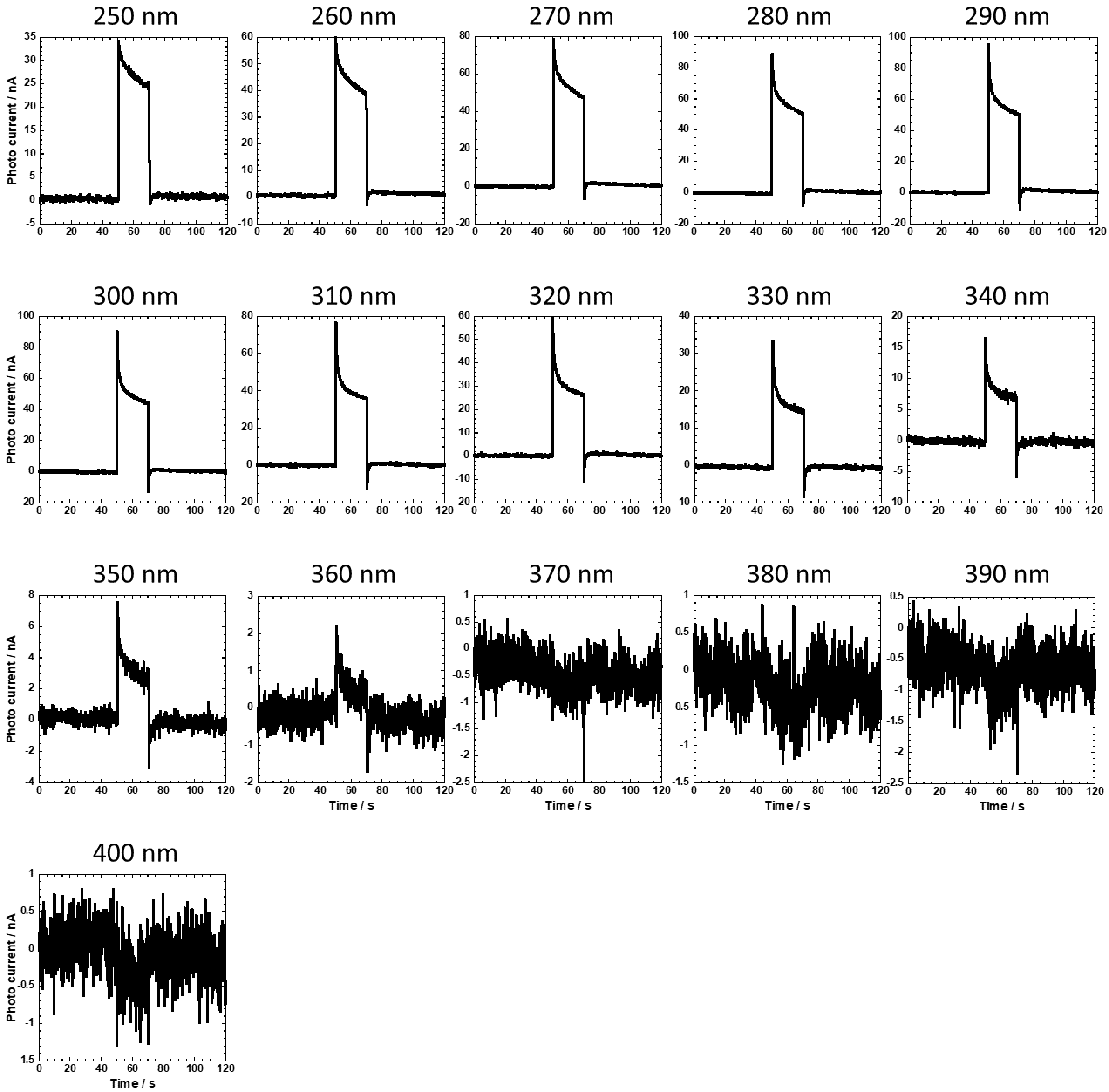

Saline_E_f 0 V_E_m 0 V

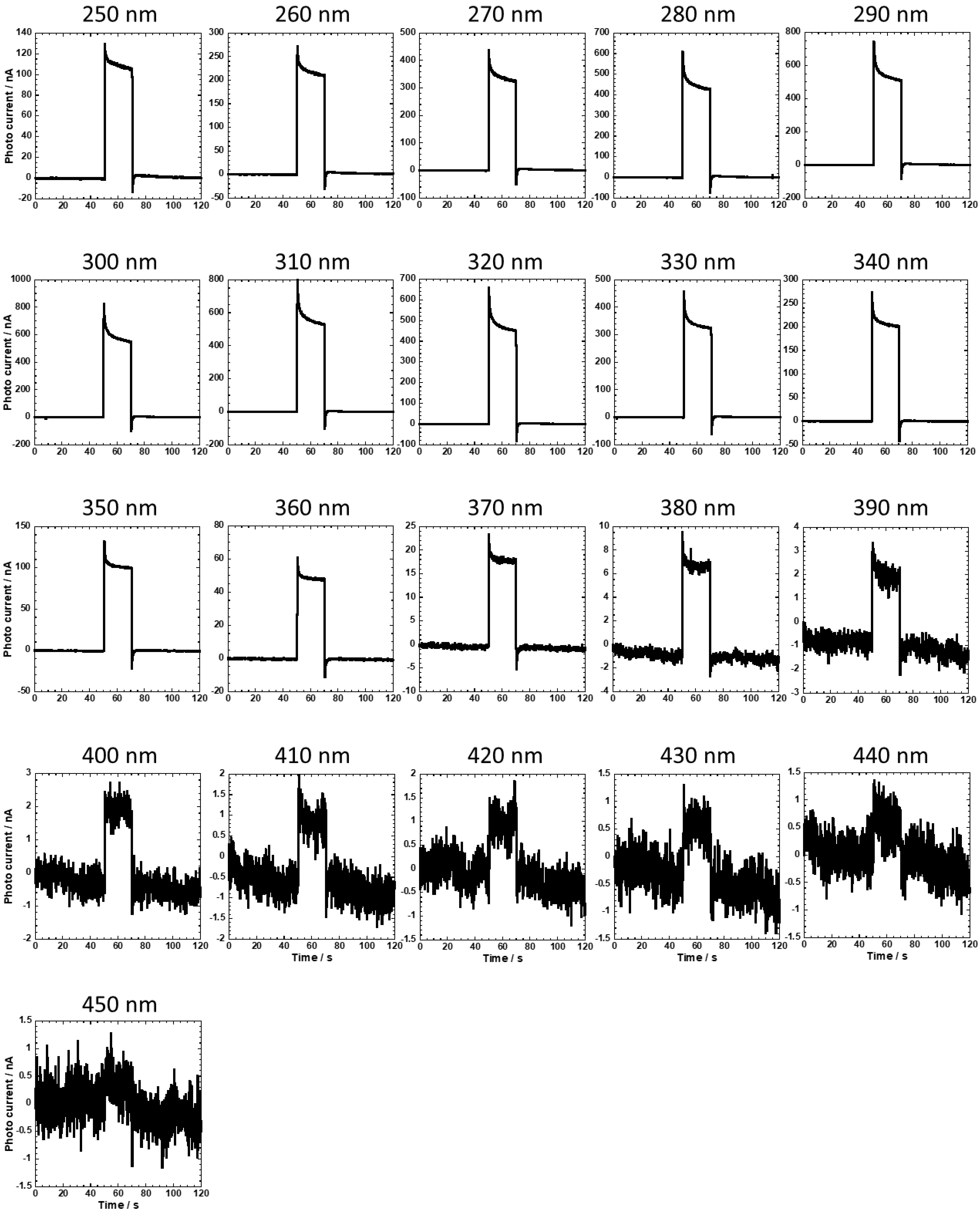

Saline_E_f 0 V_E_m -0.1 V

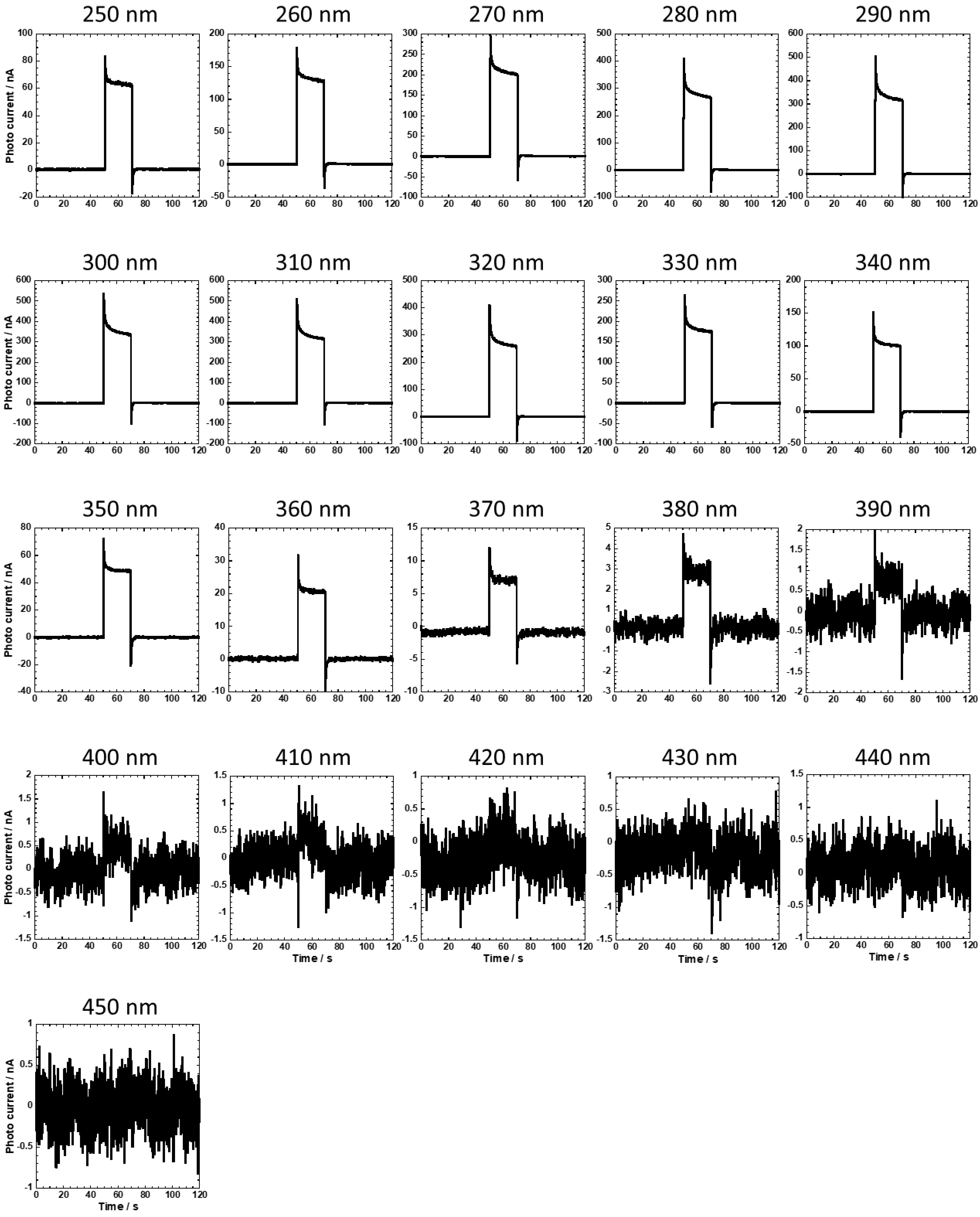

Saline_E_f 0 V_E_m -0.2 V

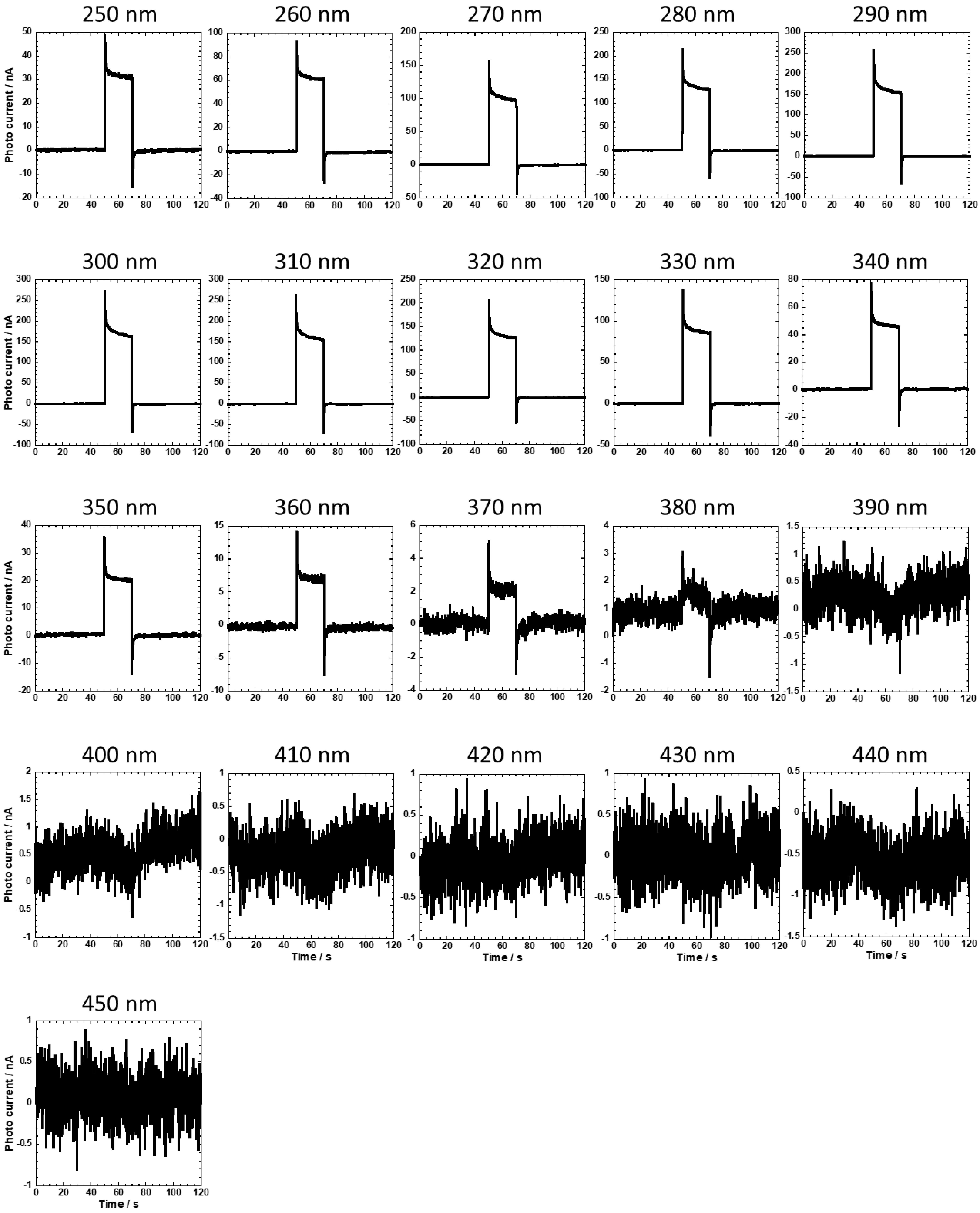

Saline_E_f -0.1 V_E_m -0.1 V

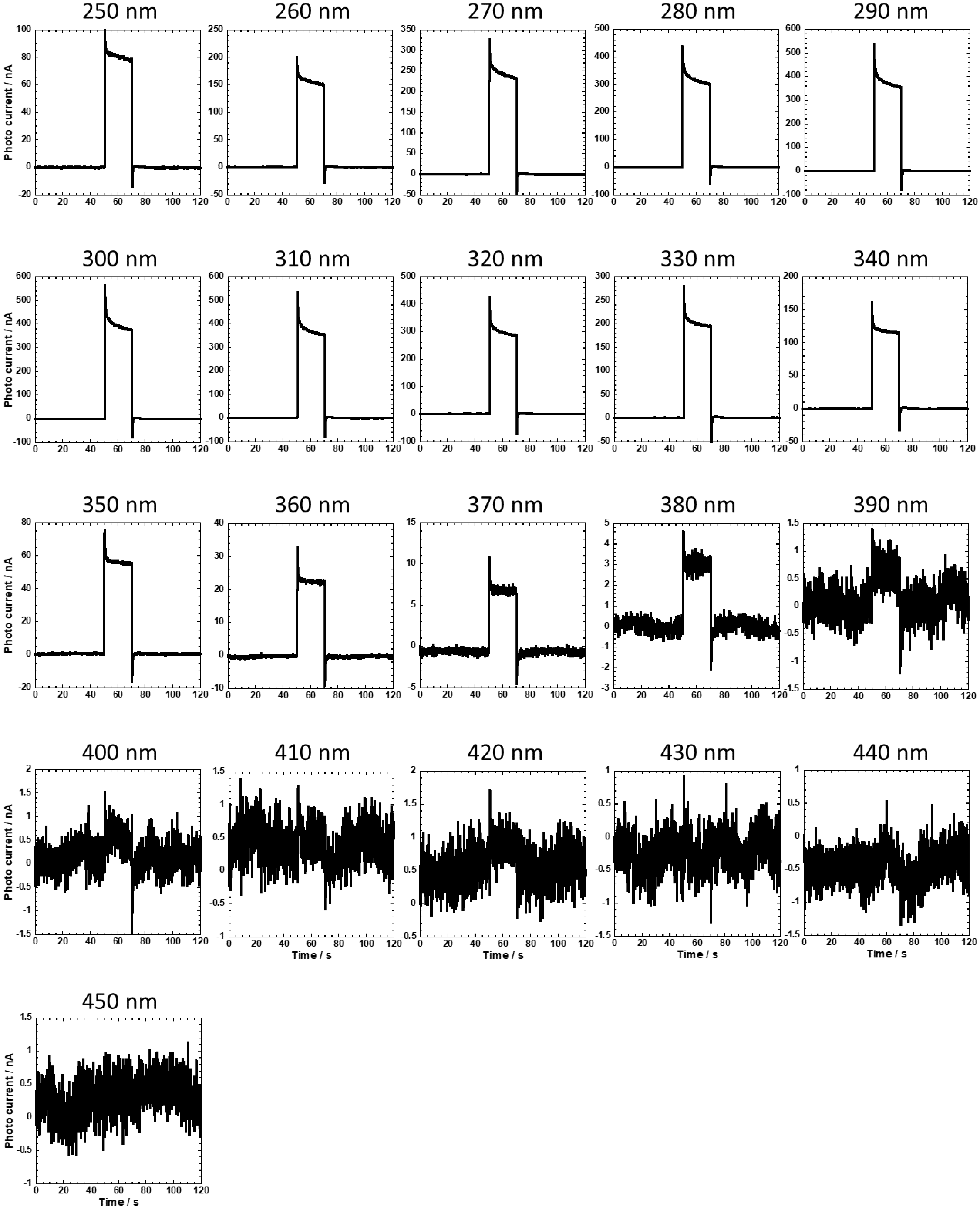

Saline_E_f -0.1 V_E_m -0.2 V

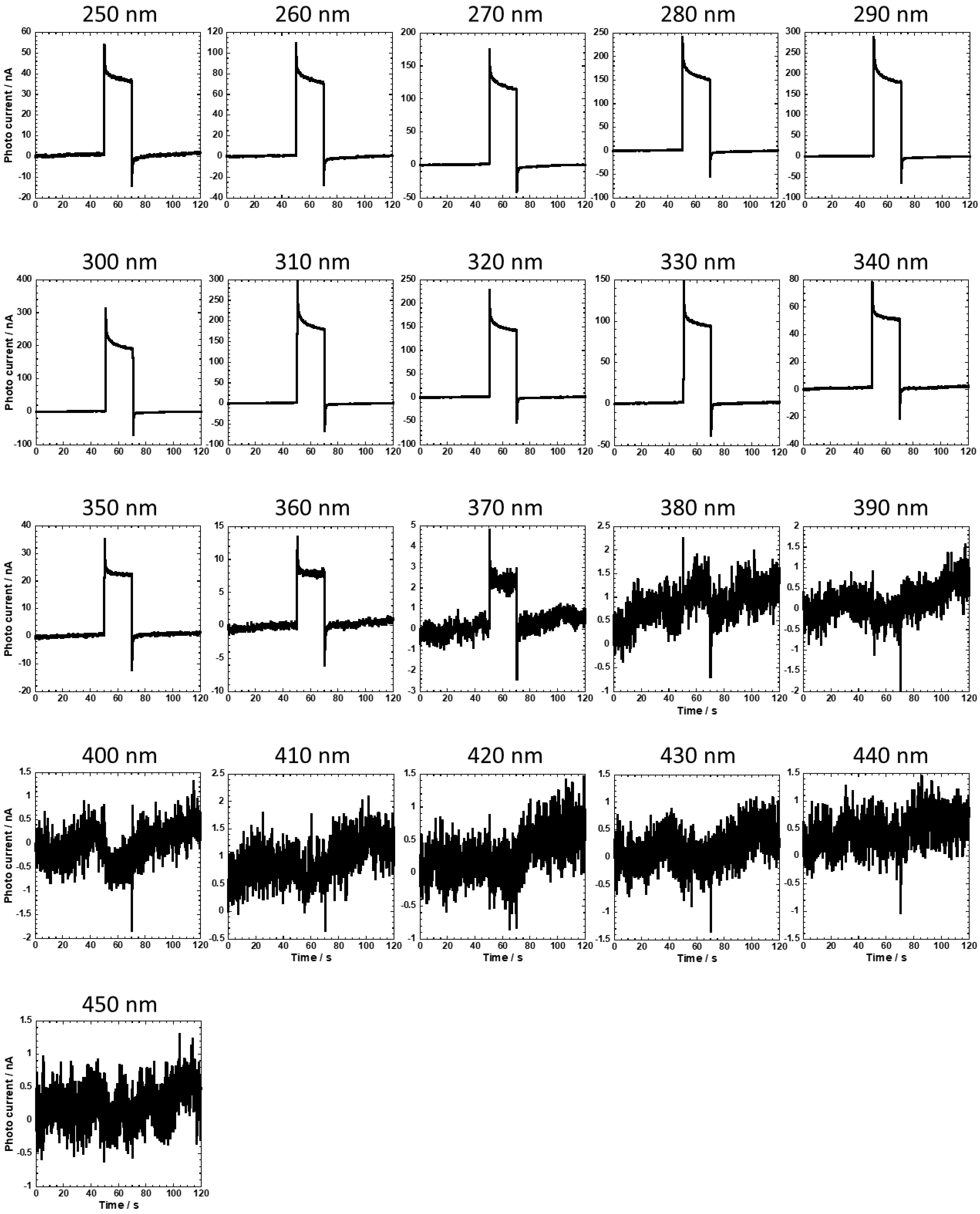

Saline_E_f -0.2 V_E_m -0.2 V

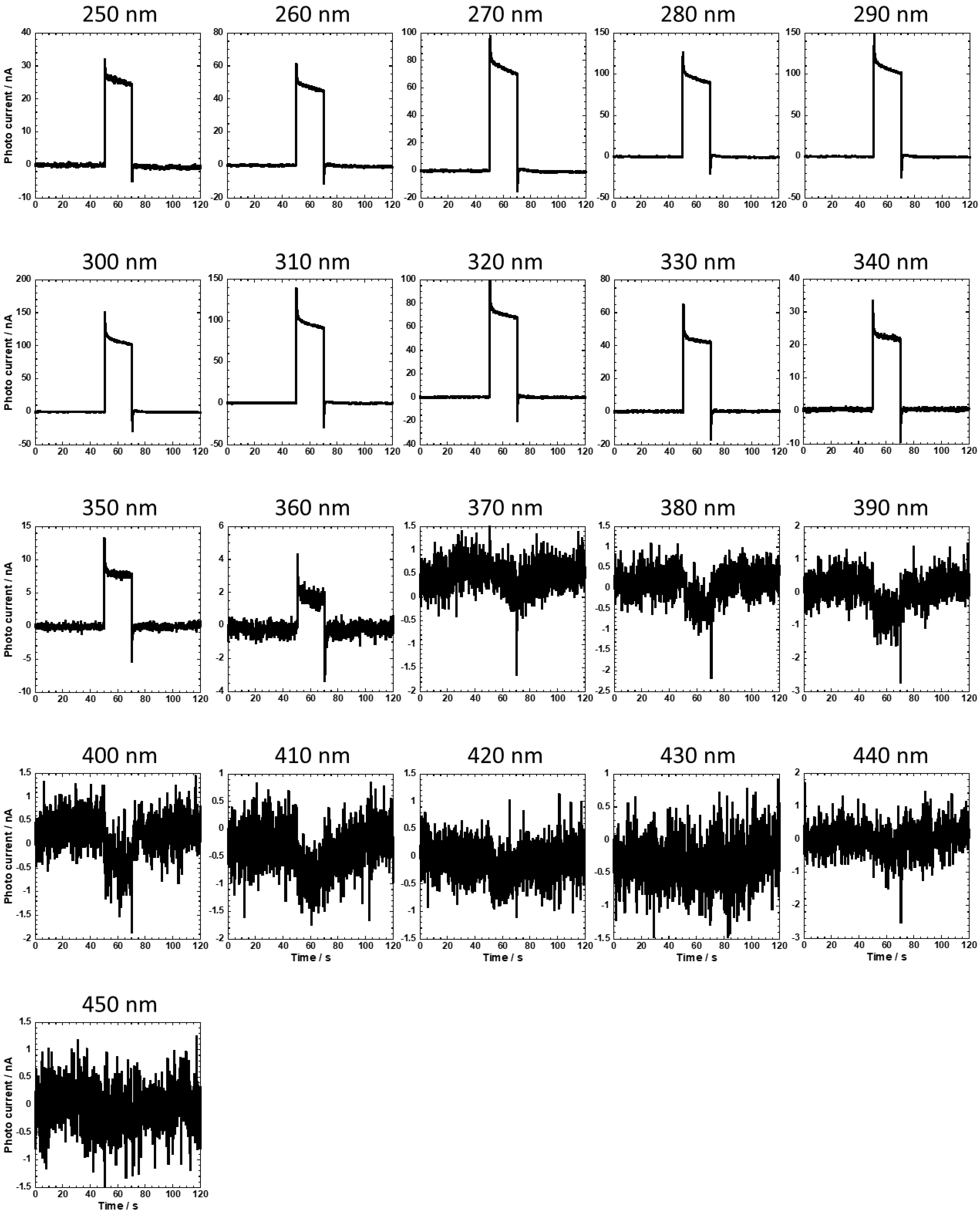

Supplement: Supplemental Material [file TSTA_A_2066960_SM7388.pdf]
