# Supplementary figures and images for: Band structures of passive films on titanium in simulated bioliquids determined by photoelectrochemical response: principle governing the biocompatibility
Source: Sci Technol Adv Mater. 2022 May 6;23(1):322–31. doi: 10.1080/14686996.2022.2066960 (PMC9090409; doi:10.1080/14686996.2022.2066960)

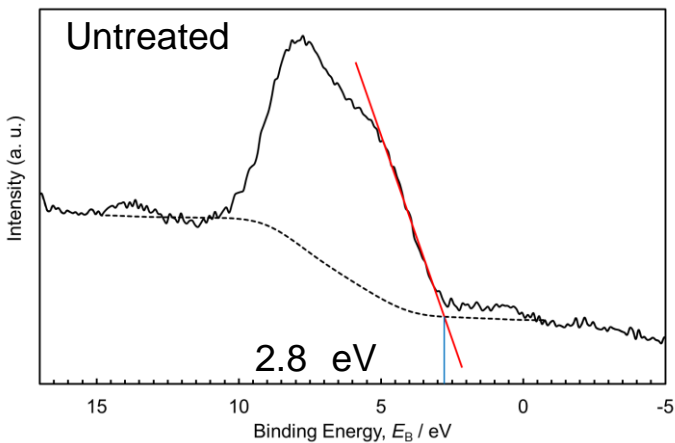

|           | -0.2 V | -0.1 V | 0 V |
|-----------|--------|--------|-----|
| Untreated | 2.8    |        |     |
| Hanks     | 2.8    | 2.9    | 2.9 |
| Saline    | 2.9    | 3.0    | 2.8 |

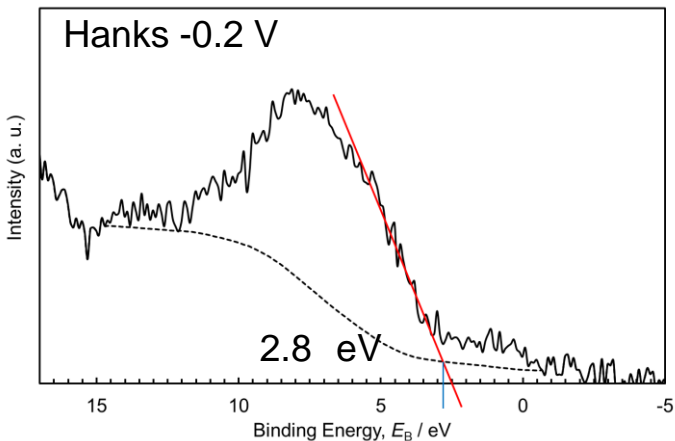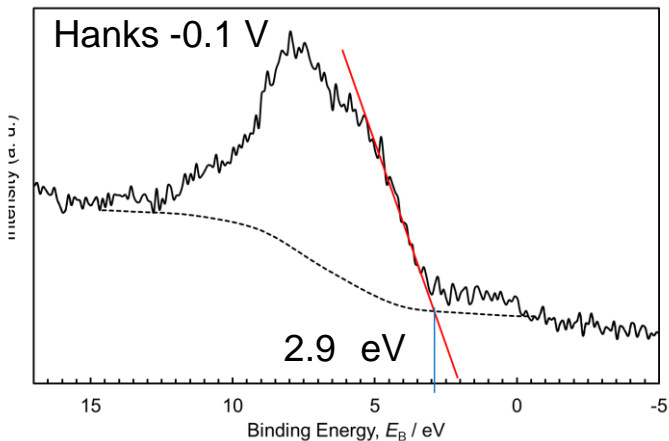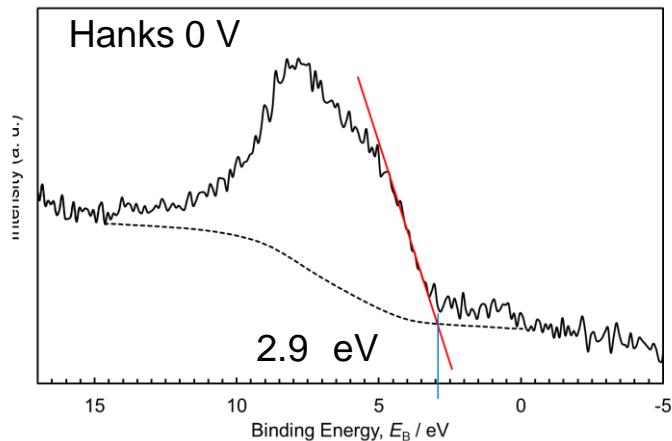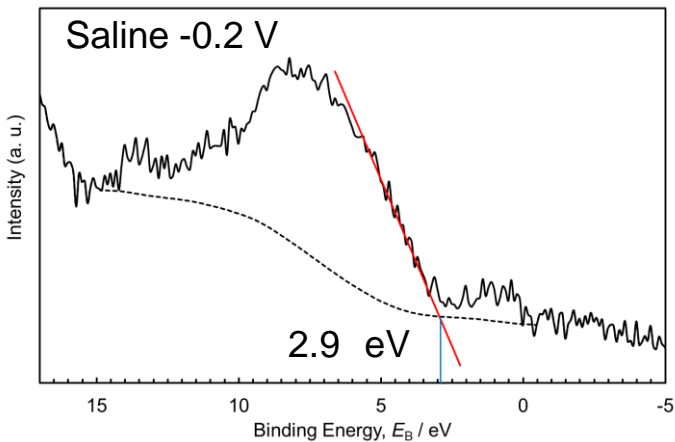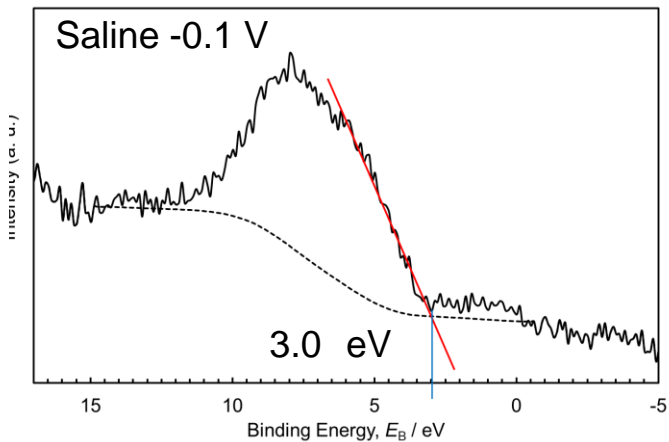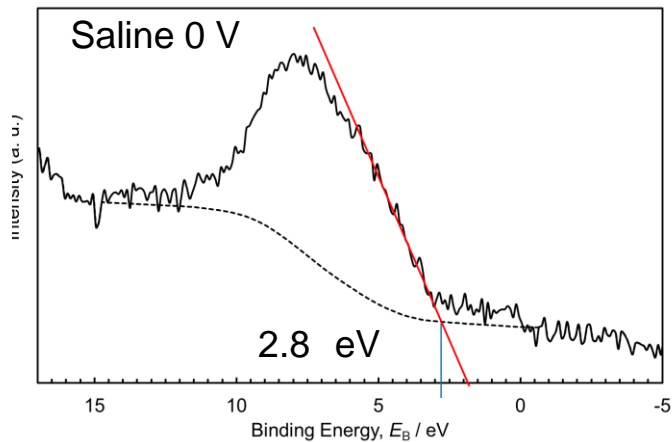

Supplement: Supplemental Material [file TSTA_A_2066960_SM7387.pdf]
