# Supplementary material for: Band structures of passive films on titanium in simulated bioliquids determined by photoelectrochemical response: principle governing the biocompatibility
Source: Sci Technol Adv Mater. 2022 May 6;23(1):322–31. doi: 10.1080/14686996.2022.2066960 (PMC9090409; doi:10.1080/14686996.2022.2066960)

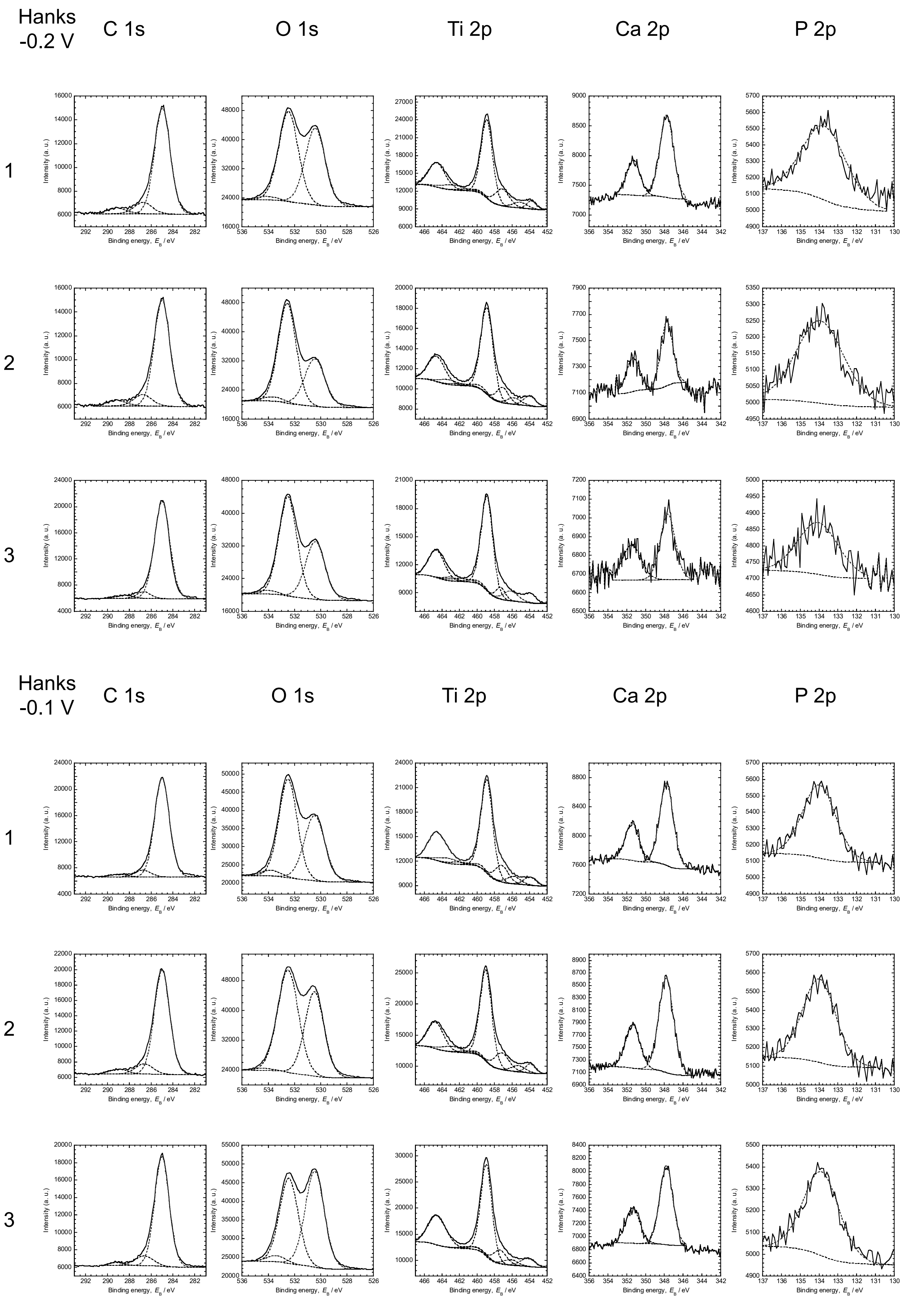
Hanks
-0.2 V
C 1s
O 1s
Ti 2p
Ca 2p
P 2p
1
2
3
Intensity (a. u.)
Binding energy, E_B / eV
Hanks
-0.1 V
C 1s
O 1s
Ti 2p
Ca 2p
P 2p
1
2
3
Intensity (a. u.)
Binding energy, E_B / eV

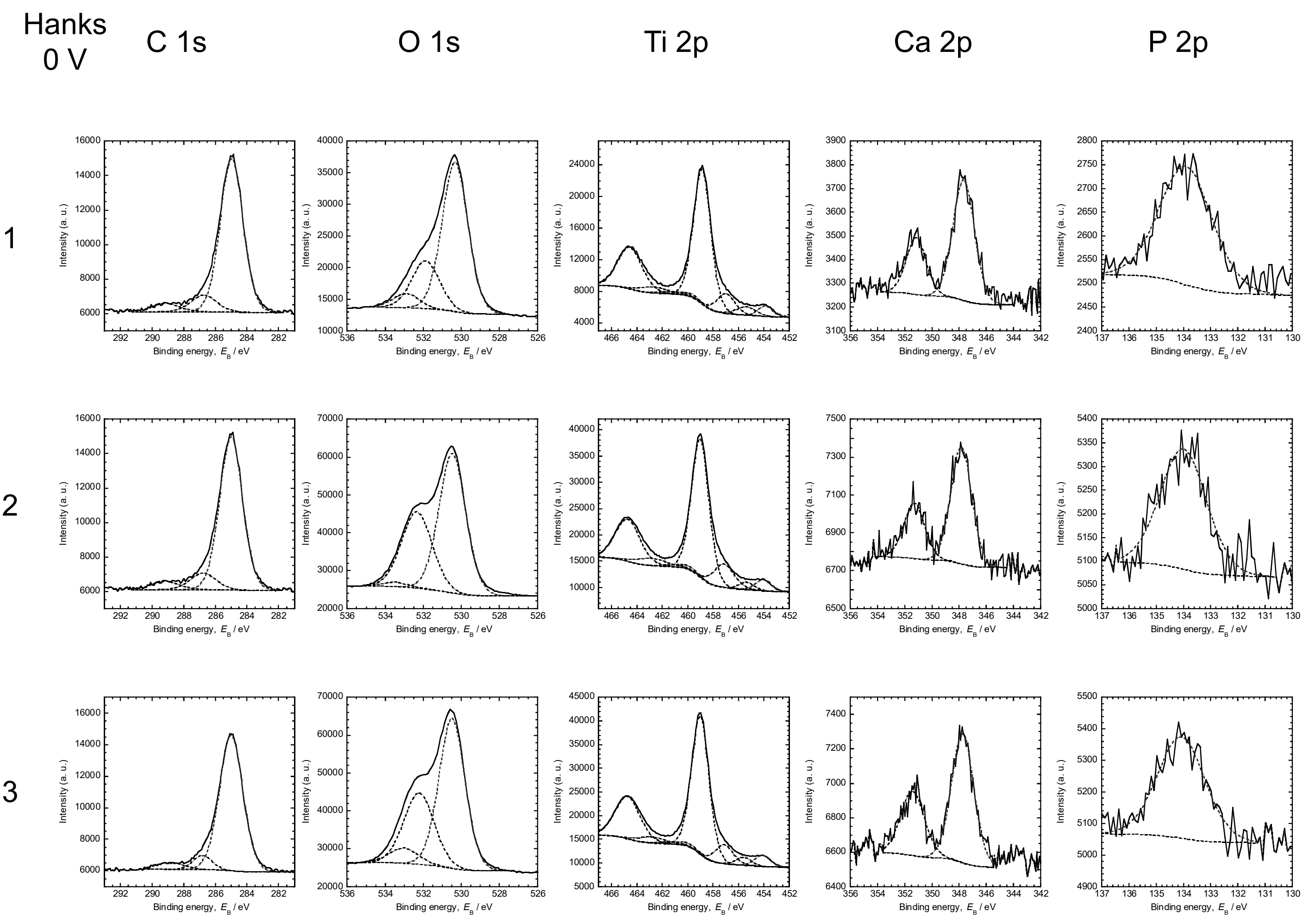
Hanks
0 V
C 1s
O 1s
Ti 2p
Ca 2p
P 2p
1
2
3
Intensity (a. u.)
Binding energy, E_B / eV

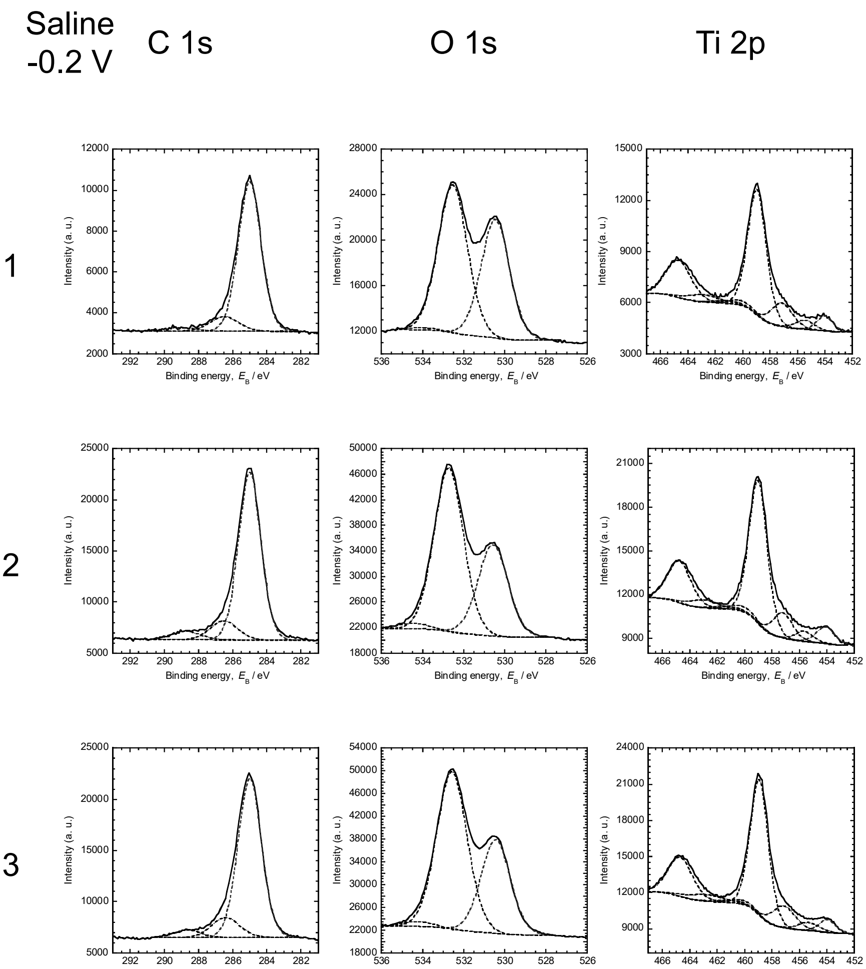
Saline
-0.2 V
C 1s
O 1s
Ti 2p
Ca 2p
P 2p
1
2
3
Intensity (a. u.)
Binding energy, E_B / eV

Saline
-0.1 V

C 1s | O 1s | Ti 2p | Ca 2p | P 2p

1

2

3

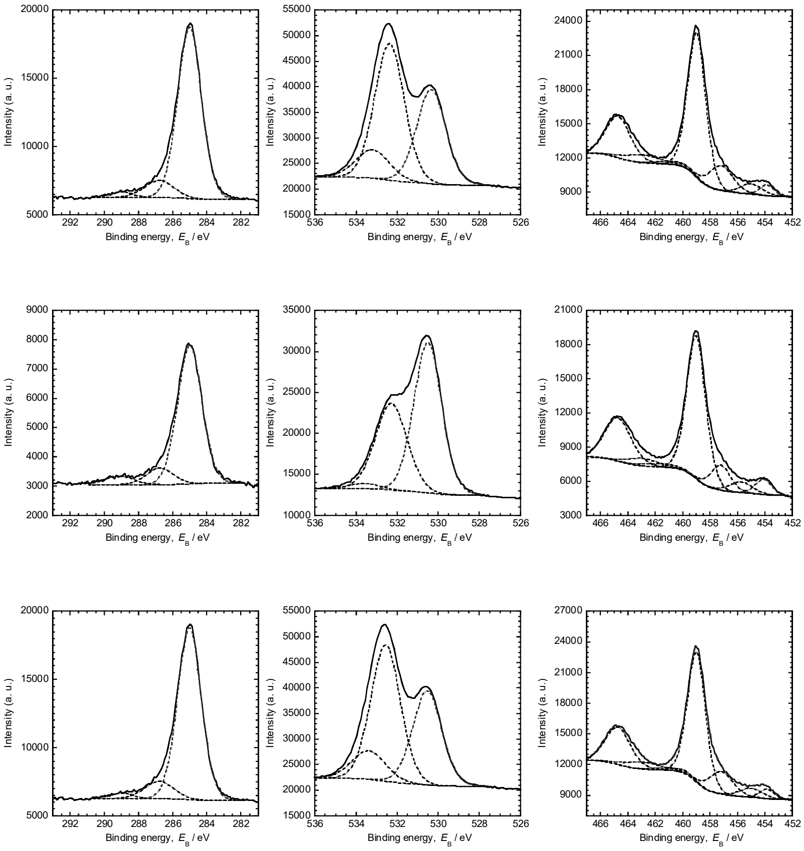

Saline
0 V

C 1s | O 1s | Ti 2p | Ca 2p | P 2p

1

2

3

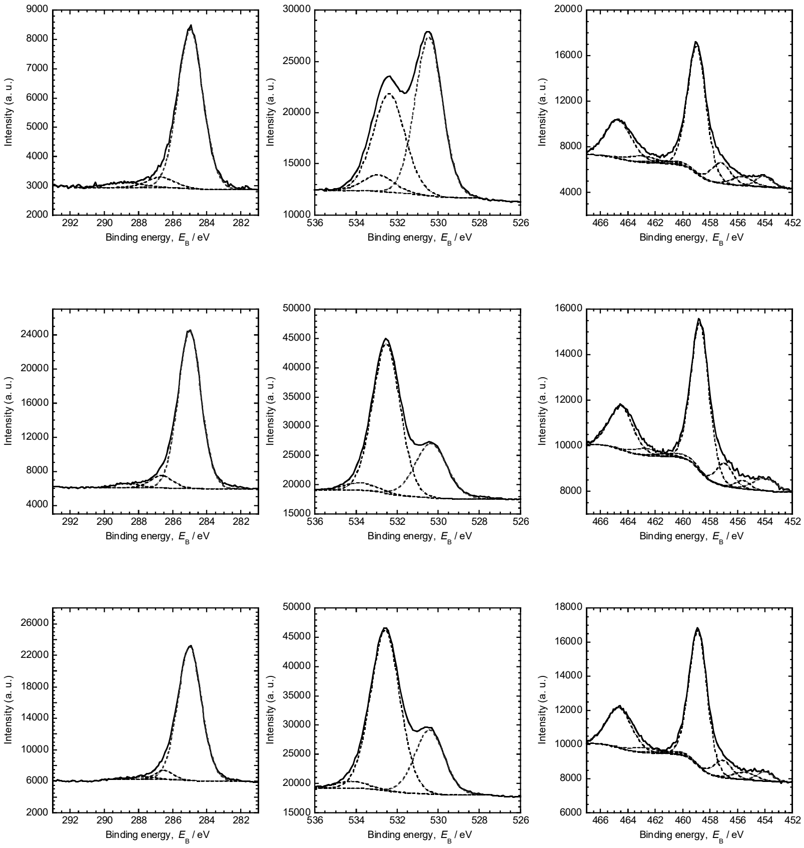

Supplement: Supplemental Material [file TSTA_A_2066960_SM7384.pdf]
